# Integrated carbon nanotube-reinforced PTFE nanofiber membranes for breathable, super-hydrophobic, and thermally resilient triboelectric nanogenerators

**DOI:** 10.1007/s42114-026-01657-2

**Published:** 2026-02-02

**Authors:** Yuxiao Wang, Lin Dong

**Affiliations:** Department of Mechanical and Industrial Engineering, New Jersey Institute of Technology, Newark, NJ 07102 USA

**Keywords:** Triboelectric nanogenerator, Multifunctional nanofiber membrane, Carbon nanotube (CNT) reinforcement, Polytetrafluoroethylene (PTFE), Breathable and superhydrophobic materials

## Abstract

The rapid advancement of self-powered wearable electronics has created an urgent demand for materials that can simultaneously enable efficient energy harvesting and reliable sensing while maintaining mechanical flexibility, breathability, and environmental robustness. Here, a carbon nanotube (CNT)-reinforced polytetrafluoroethylene (PTFE) nanofiber membrane with multiple functionalities is developed through an electrospinning-sintering strategy, integrating high breathability, super-hydrophobicity, and thermal resilience within a single architecture. The incorporation of CNTs enhances dielectric properties and mechanical strength, forming a robust charge-retentive fibrous network. The optimized membrane exhibits a super-hydrophobic surface with a water contact angle of 155.49°, along with excellent air permeability and outstanding thermal stability. When assembled into a triboelectric nanogenerator (TENG), the device delivers an open-circuit voltage of ~ 122 V, sufficient to power portable electronics. The TENG also demonstrates excellent fouling resistance and self-cleaning capability, maintaining stable performance after repeated contamination-washing cycles. Owing to its breathable and flexible configuration, the device enables real-time motion and respiration monitoring without compromising air exchange. It also exhibits consistent output under high-temperature (up to 250 °C) and underwater conditions, with the latter further showcased through Morse-code communication. This work establishes a scalable materials design strategy for multifunctional nanofibrous TENGs, offering a versatile platform for adaptive, self-powered electronic systems operable across diverse environments.

**Supplementary Information:**

The online version contains supplementary material available at 10.1007/s42114-026-01657-2.

## Introduction

The rapid advancement of wearable electronics and intelligent health-monitoring systems has spurred an urgent demand for flexible energy harvesters and self-powered sensors that can operate continuously without reliance on external power supplies [1–4]. Triboelectric nanogenerators (TENGs) can convert ubiquitous mechanical stimuli into electrical energy via coupled triboelectrification and electrostatic induction [5]. Accordingly, they have emerged as a promising technology for realizing sustainable energy solutions and self-powered electronic systems. Conventional piezoresistive and capacitive sensors require continuous electrical power and are often susceptible to environmental fluctuations, while piezoelectric and electromagnetic generators are typically constrained by low voltage output or bulky, rigid architectures [6–8]. In contrast, TENGs provide intrinsic energy autonomy combined with high voltage output, lightweight configuration, and broad adaptability to diverse mechanical and environmental conditions, enabling seamless integration into next-generation wearable platforms [9, 10]. In particular, the broad material compatibility of TENGs enables customized designs for diverse applications, such as highly stretchable elastomers for conformal wearables or micro/nanostructured hydrophobic surfaces for enhanced output and durability [11, 12]. However, as TENGs are increasingly deployed in complex and harsh environments, the triboelectric layer materials are expected to possess not only high charge-generation capability but also multifunctional robustness. Despite extensive progress, most existing material systems can fulfill certain performance requirements only by compromising others. For instance, triboelectric materials engineered for high electrical output often exhibit poor permeability, whereas those designed for thermal robustness frequently sacrifice hydrophobicity or flexibility [13, 14]. Achieving these distinct functionalities within a single integrated material platform remains a significant challenge. These persistent trade-offs underscore the critical need for innovative design strategies that enable truly multifunctional triboelectric materials. Such materials should concurrently deliver high breathability, super hydrophobicity, and thermal resilience, attributes that have seldom been achieved simultaneously in current systems.

Among these attributes, breathability plays a crucial yet often overlooked role. Insufficient air permeability leads to the accumulation of heat and moisture at the skin-device interface, causing discomfort during prolonged use. Although porous and sponge-structured TENGs have been widely developed to improve air permeability and mechanical compliance, they still face inherent challenges related to structural stability and environmental susceptibility [15, 16]. For example, the large and irregular pores of microporous sponge-based TENGs may readily absorb moisture and trap dust, leading to charge dissipation and reduced operational stability. Moreover, their soft frameworks are prone to mechanical fatigue or collapse under cyclic deformation, resulting in fluctuating electrical output. Textile-based TENGs possess intrinsic softness, flexibility, and permeability derived from woven or knitted microstructures, enabling comfortable and conformal integration with garments [17, 18]. Nevertheless, the addition of metallic electrodes, encapsulation films, or hydrophobic coatings frequently obstructs pore channels and restricts air exchange, thereby diminishing the inherent breathability. These contradictions highlight the need for breathable triboelectric layers that strike an optimal balance between porosity, durability, and charge-generation ability, ensuring reliable long-term operation.

In addition to air breathability, hydrophobicity and self-cleaning capability are equally essential for the reliable operation of TENGs. In practical environments, sweat, dust, and humidity can easily contaminate triboelectric surfaces, altering surface charge states and deteriorating electrical output performance. Super-hydrophobic surfaces may provide a natural means of mitigating fouling by repelling water and enabling contaminants to roll off. To achieve enhanced hydrophobicity, many studies have employed fluorinated coatings on triboelectric layers; however, such coatings inevitably compromise material breathability, exhibit poor adhesion, and involve complex fabrication processes [19]. Alternatively, micro/nanostructured surfaces have been introduced to impart hydrophobicity, but these fragile textures often lack long-term mechanical robustness under repeated frictional contact [20]. Once damaged, the hydrophobic layer rapidly loses its functionality, leading to irreversible performance degradation. Developing durable super-hydrophobic interfaces is crucial for constructing self-maintaining TENGs. By integrating low surface energy with mechanical toughness, these interfaces ensure stable operation and a long lifespan under prolonged cycling and environmental exposure.

Ensuring stable operation of TENGs in real-world environments requires not only hydrophobic protection against moisture and contaminants but also thermal stability to withstand elevated or fluctuating temperatures. Most polymer-based TENGs suffer from low melting points and poor dimensional stability, restricting their use to ambient conditions. High-temperature scenarios, such as those encountered in industrial monitoring, firefighting, or desert environments, require triboelectric materials that can maintain both structural integrity and charge-generation ability under thermal conditions [14, 21]. Existing heat-resistant polymers, such as liquid crystalline polymer (LCP), polyimide (PI), and aramid, often exhibit poor breathability or pronounced moisture uptake, which can compromise triboelectric performance and limit their suitability for breathable wearable electronics or outdoor applications [22–24]. To realize truly multifunctional and environment-adaptive TENGs, it is imperative to engineer triboelectric materials that integrate breathability, super-hydrophobicity, and thermal robustness within a single framework. However, achieving this combination remains challenging and has been seldom reported.

Here, we report a high-performance carbon nanotube (CNT)-reinforced PTFE nanofiber membrane that integrates breathability, super-hydrophobicity, and thermal resilience within a single architecture for multifunctional TENG applications. CNTs were incorporated into the PTFE matrix through a high-temperature melting-fusion mechanism. This approach avoids complex surface-functionalization while enhancing the dielectric properties and mechanical integrity of the fibrous network, thereby boosting the overall electrical output of the TENG device. The electrospinning-sintering strategy transforms PTFE nanoparticles into a continuous fibrous network with intrinsic permeability and mechanical flexibility. As a result, the user comfort and long-term wearability of the TENG device are improved. The combination of low surface energy and hierarchical micro/nanostructures yields a superhydrophobic surface with a water contact angle of 155.49°. This endows the TENG device with self-cleaning functionality and reliable operation even under underwater conditions. Furthermore, the inherent thermal robustness of PTFE endows the TENG with reliable operation and consistent output even under high-temperature environments. This work presents an integrated and scalable materials design strategy for constructing multifunctional PTFE-based triboelectric membranes. The resulting architecture effectively bridges energy harvesting, self-powered sensing, and adaptive operation while balancing comfort, resilience, and performance. Overall, the proposed strategy advances the practical deployment of TENGs in next-generation wearable and extreme-environment electronic systems.

## Results and discussion

### Rational design of multifunctional PTFE-based TENGs

Among the various triboelectric materials investigated, polytetrafluoroethylene (PTFE) has long been recognized as a representative high-performance candidate owing to its strong electronegativity and remarkable environmental stability [25]. These properties originate from its linear -(CF₂-CF₂)- backbone, which endows PTFE with outstanding chemical inertness, thermal robustness, and intrinsically low surface energy, making it particularly suitable for durable triboelectric interfaces. Nevertheless, conventional PTFE-based TENGs typically utilize dense commercial PTFE films fabricated through a calendering process or PTFE blended composites, which renders these devices impermeable and prone to heat accumulation and discomfort in wearable applications [26–29]. Moreover, to enhance electrical output, these films often require additional surface modification treatments, such as plasma etching or ion implantation, which increases fabrication complexity and limits scalability [30, 31]. Electrospinning has recently been explored to construct porous PTFE nanofiber membranes with excellent breathability and enlarged surface area, with the latter providing more effective contact interfaces for improved triboelectric charge generation [32–34]. However, prior studies have primarily focused on output enhancement while overlooking PTFE’s intrinsic advantages of high-temperature tolerance and hydrophobicity, confining their applications to ambient environments. These limitations highlight the need to develop multifunctional PTFE-based triboelectric membranes that can simultaneously deliver high output performance, mechanical flexibility, air permeability, thermal robustness, and self-cleaning capability.

**Fig. 1:**
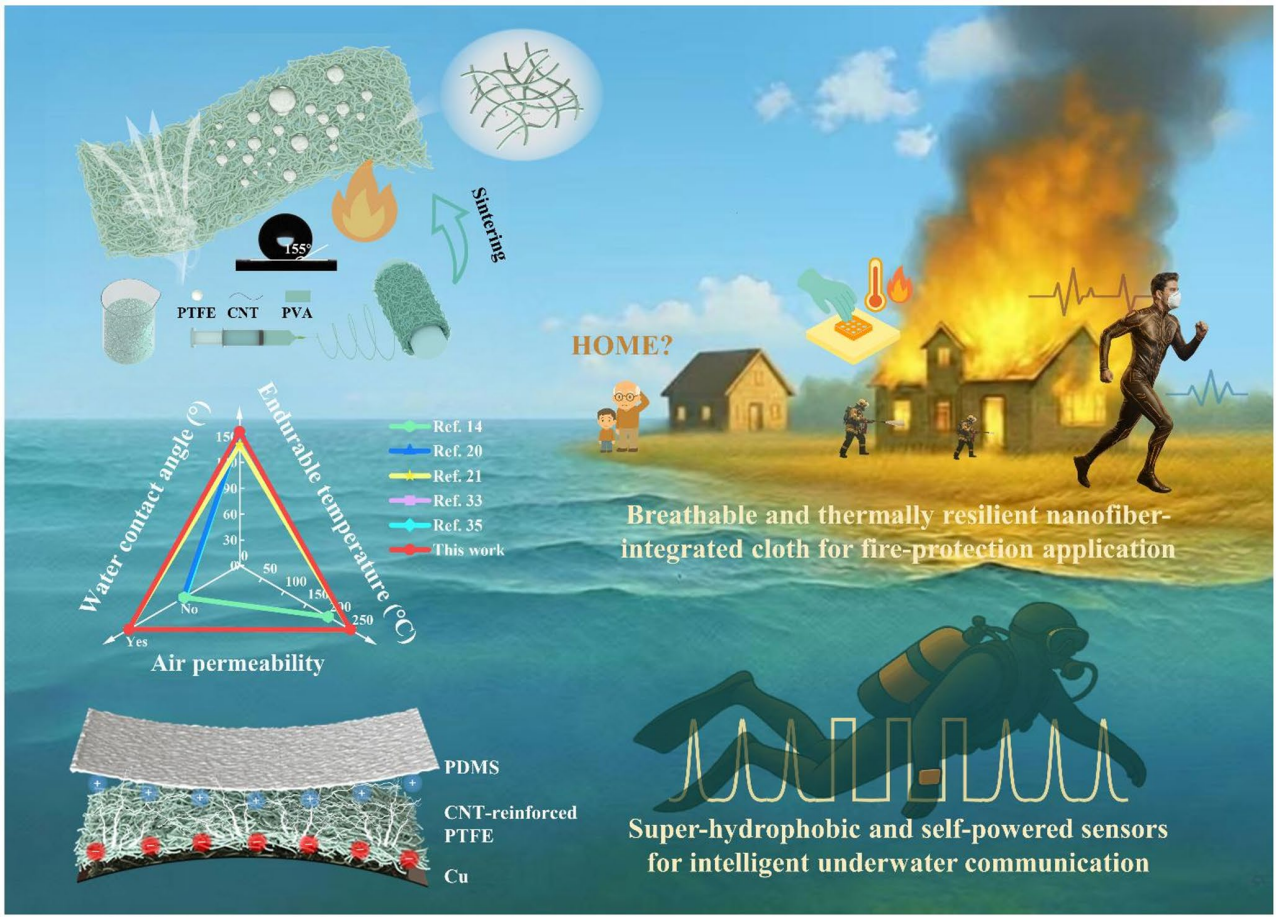
Schematic illustration of the CNT-reinforced PTFE-based TENG fabricated via an electrospinning-sintering strategy, integrating material design, performance optimization, structural configuration, and multifunctional applications in energy harvesting and self-powered sensing under high-temperature and underwater conditions

To meet these stringent demands, an integrated design strategy was proposed in this work, as illustrated in Fig. 1, which combines materials design, performance optimization, structural configuration, and multifunctional application into a unified framework. The core component of this system is the CNT-reinforced PTFE nanofiber membrane fabricated through an electrospinning-sintering route. In this process, an aqueous PTFE dispersion was co-electrospun with PVA serving as a sacrificial template to provide chain entanglement and spinnability, while CNTs were incorporated as conductive fillers to enhance the dielectric constant and charge storage capability. The subsequent high-temperature sintering of the as-spun composite nanofibers ensured complete removal of PVA and fusion of PTFE nanoparticles into a continuous fibrous network. The resulting membrane exhibits high flexibility, excellent air permeability, outstanding thermal stability, and a water contact angle of 155.49°, indicating super-hydrophobicity. These characteristics collectively provide an ideal foundation for constructing multifunctional TENGs operable under diverse environmental conditions. A photograph of the as-prepared flexible CNT-reinforced PTFE nanofiber membrane is provided in Figure S1. To evaluate the comprehensive performance advantages of the electrospun PTFE membrane, three key parameters-air permeability, water contact angle, and endurable temperature-were compared against other references [14, 20, 21, 33, 35]. As summarized in the radar chart in Fig. 1, the PTFE nanofiber membrane simultaneously achieves superior air permeability and thermal endurance, while maintaining superior super-hydrophobicity. Such a balanced combination of breathability and robustness effectively addresses the conventional trade-offs encountered in flexible energy harvesting materials, where enhanced breathability often compromises environmental stability.

Constructed from a super-hydrophobic PTFE nanofiber membrane, a microstructured PDMS counter layer, and a copper electrode, the PTFE-based TENG forms a flexible and robust triboelectric interface capable of efficient energy harvesting and adaptive sensing. During operation, periodic contact-separation between the PTFE and PDMS layers induces triboelectric charges through the coupling of contact electrification and electrostatic induction, thereby generating electrical energy. The porous and roughened surface structure not only increases the effective contact area but also promotes gas exchange across the membrane, ensuring mechanical adaptability and comfort when used in wearable configurations. Benefiting from these rational materials and device design, the PTFE-based TENG demonstrates versatile functionality across multiple scenarios. The device effectively powers small-scale electronics, including a digital wristwatch, thermo-hygrometer, and LED array, demonstrating its capability for self-sustained energy harvesting. Beyond powering electronics, the TENG can conformally attach to various body parts for self-powered motion and respiration monitoring, where distinct voltage patterns correspond to different motion intensities and breathing behaviors. Furthermore, leveraging the intrinsic super-hydrophobicity and thermal stability of PTFE, the device maintains stable performance under high-temperature (up to 250 °C), underwater, and ambient conditions. Overall, this integrated design, combining electrospun PTFE nanofiber membranes, CNT-enhanced dielectric properties, and hierarchical device architecture, establishes a multifunctional, breathable, and environmentally resilient TENG platform. The system seamlessly bridges energy harvesting, wearable sensing, and intelligent signaling, providing a scalable and robust foundation for next-generation self-powered electronic systems.

### Thermal robustness, high permeability, and super-hydrophobicity

**Fig. 2:**
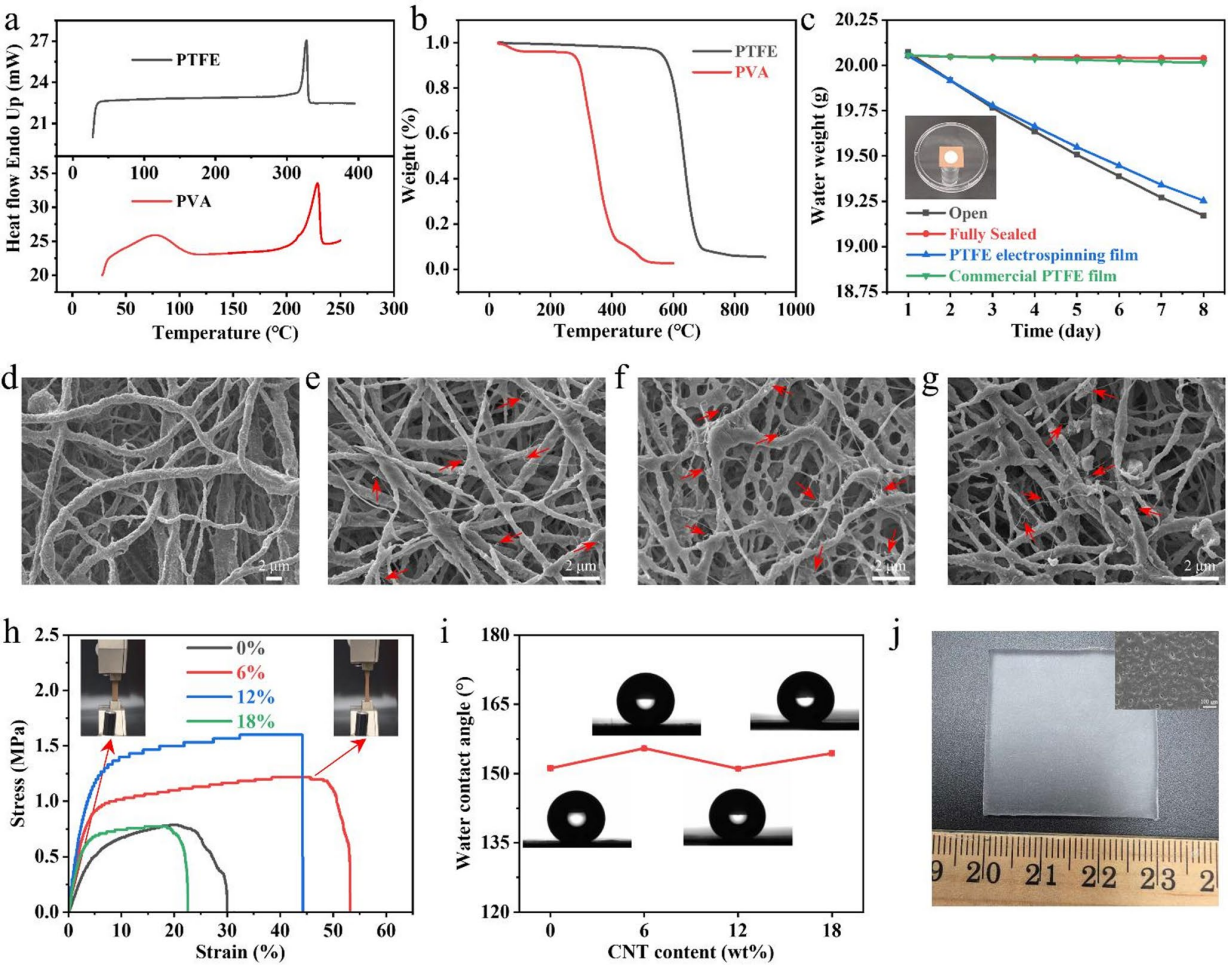
Material characterization of the PTFE and PVA materials. (**a**) DSC and (**b**) TGA curves of PTFE and PVA. (**c**) Air permeability of PTFE nanofiber membrane compared with a commercial PTFE membrane. (**d**-**g**) Field emission scanning electron microscope (FE-SEM) images of PTFE nanofiber membranes with different CNT loadings (**d**: 0 wt%, e: 6 wt%, f: 12 wt%, g: 18 wt%). Representative CNTs are highlighted by arrows for clarity. (**h**) Tensile stress-strain curves of PTFE nanofiber membranes with varying CNT contents. (**i**) Water contact angles of PTFE nanofiber membranes with different CNT loadings. (**j**) Photograph and SEM images of the PDMS to show the rough surface of PDMS film

PTFE is renowned for its exceptional chemical inertness, thermal stability, and electrical insulation, making it well suited for high-temperature and corrosive environments. However, PTFE resin is essentially insoluble in common solvents and exhibits very low electrical conductivity, rendering direct electrospinning infeasible. Although supplied in aqueous dispersion form, PTFE remains as discrete colloidal particles instead of molecularly dissolved chains, preventing the formation of an entangled polymer network and limiting the viscoelasticity needed for electrospinning. To render PTFE electrospinnable, PVA was used in this research as a sacrificial template to supply chain entanglement and increase the viscoelasticity and spinnability. After co-electrospinning with the aqueous PTFE dispersion, a subsequent sintering step removed PVA and promoted PTFE particle coalescence into a continuous nanofibrous membrane. To define a sintering window that ensures complete PVA decomposition while enabling PTFE melting without degradation, the thermal behaviors of PTFE and PVA were characterized by DSC and TGA (Fig. 2a, b). The DSC curve of PTFE particles shows a sharp endothermic transition at around 327 °C, corresponding to the crystalline melting point of PTFE. In contrast, PVA powders exhibit two distinct endothermic peaks: the first at ~ 76 °C, which is attributed to the evaporation of absorbed water molecules, as the abundant hydroxyl groups in PVA readily form hydrogen bonds with water and thereby facilitate moisture uptake, and the second at ~ 229 °C, corresponding to the melting of the PVA matrix. TGA further revealed the different decomposition behaviors of the two components. PVA starts to degrade at ~ 292 °C, with the maximum degradation rates occurring at 312 °C and 359 °C. Based on the decomposition temperature of PVA and the melting point of PTFE, the sintering temperature was set at 375 °C. This temperature is sufficient to eliminate the majority of PVA while simultaneously inducing melting and inter-fiber fusion of PTFE, thereby enhancing the mechanical integrity of the nanofiber membrane without inducing polymer degradation. The TGA curve of the PTFE nanofiber membrane further shows that PTFE remains stable up to ~ 592 °C, confirming its high thermal stability. In addition to thermal stability, the gas permeability of the membranes was also examined (Fig. 2c). The bottle sealed with a commercial PTFE membrane exhibited negligible water weight loss, indicating its impermeable nature. In contrast, the PTFE electrospinning nanofiber membrane allowed a weight change comparable to that of the uncovered bottle, demonstrating excellent breathability. This superior permeability is attributed to the interconnected porous structure of the electrospinning membranes, which enables efficient vapor transport. The inset in Fig. 2c further visualizes this effect, showing that water vapor from a bottle containing hot water at 90 °C can readily and rapidly pass through the PTFE nanofiber membrane.

CNTs are highly conductive carbon-based nanofillers with ultrahigh aspect ratios, capable of forming percolative networks at low loadings. When incorporated into aqueous PTFE dispersions, CNTs can increase precursor conductivity, stabilizing the electrospinning jet and promoting uniform, continuous fiber formation. Within the membrane, well-dispersed CNTs can embed in or bridge fibers to create potential conductive pathways and enhance load transfer after sintering. To clarify the distribution of CNTs within the PTFE fibers, the SEM morphologies of membranes prepared with different CNT loadings were examined (Fig. 2d-g). After sintering, the majority of the PVA sacrificial component was removed, and the PTFE particles fused into continuous fibrous structures. In addition, adhering inter-fiber junctions were formed at the contact points, which are expected to enhance the mechanical integrity of the membranes. Compared with the pristine PTFE membrane, the incorporation of 6 wt% CNTs (Fig. 2e) led to CNTs being clearly embedded within the PTFE fibers. Some CNTs were anchored at one end to the PTFE matrix while the remaining segments extended along the fiber surface or into the pores, whereas others were predominantly encapsulated inside the fibers with only small fragments exposed on the surface. This hybrid distribution provides both internal mechanical reinforcement and potential conductive pathways across the fibrous network. When the CNT content was further increased to 12 wt% (Fig. 2f), a substantially higher density of CNTs was observed. In many regions, CNTs intercrossed with each other, forming continuous conductive pathways throughout the fibrous network. However, at a CNT loading of 18 wt% (Fig. 2g), the CNT distribution became markedly less uniform. Distinct PTFE agglomerates were also detected, which can be attributed to excessive CNT incorporation leading to strong entanglement between CNTs and PTFE particles. This entanglement hinders the stretching and alignment of fibers during electrospinning, ultimately resulting in heterogeneous morphology and compromised structural uniformity. Such structural evolution is directly correlated with the tensile performance of the membranes. As shown in Fig. 2h, the tensile stress increased progressively as the CNT content rose from 0 to 12 wt%. This improvement originates from the reinforcing effect of CNTs embedded within the PTFE fibers, analogous to the role of steel bars in reinforced concrete, thereby strengthening the fibrous network. However, when the CNT content was further increased to 18 wt%, the stress decreased markedly due to CNT entanglement and the formation of PTFE agglomerates, which compromised the structural uniformity and mechanical integrity of the membranes.

Furthermore, the surface wettability of the membranes was further evaluated by water contact angle measurements (Fig. 2i). All nanofiber membranes exhibited contact angles greater than 150°, confirming their super-hydrophobic nature. In particular, the PTFE membrane containing 6 wt% CNTs reached a maximum contact angle of 155.49°. The inherent low surface energy of PTFE, together with the micro/nanoscale roughness on the PTFE fiber surface, effectively facilitated the formation of the super-hydrophobicity, while the incorporation of CNTs did not exert a significant influence on the wettability. Such outstanding water repellency is expected to impart excellent self-cleaning capability to the membranes, thereby greatly extending their operational durability. In this work, PDMS film was selected as the counter triboelectric layer to pair with the electrospun PTFE membrane, owing to its inherent flexibility, facile processability, and good biocompatibility. Notably, during fabrication, sandpaper was used as the casting substrate to impart micro-scale surface roughness to the PDMS film, which is expected to further enhance the triboelectric output by increasing the effective contact area. A representative photograph and SEM images of the as-prepared PDMS film are shown in Fig. 2j.

### Energy harvesting and self-cleaning performance

**Fig. 3:**
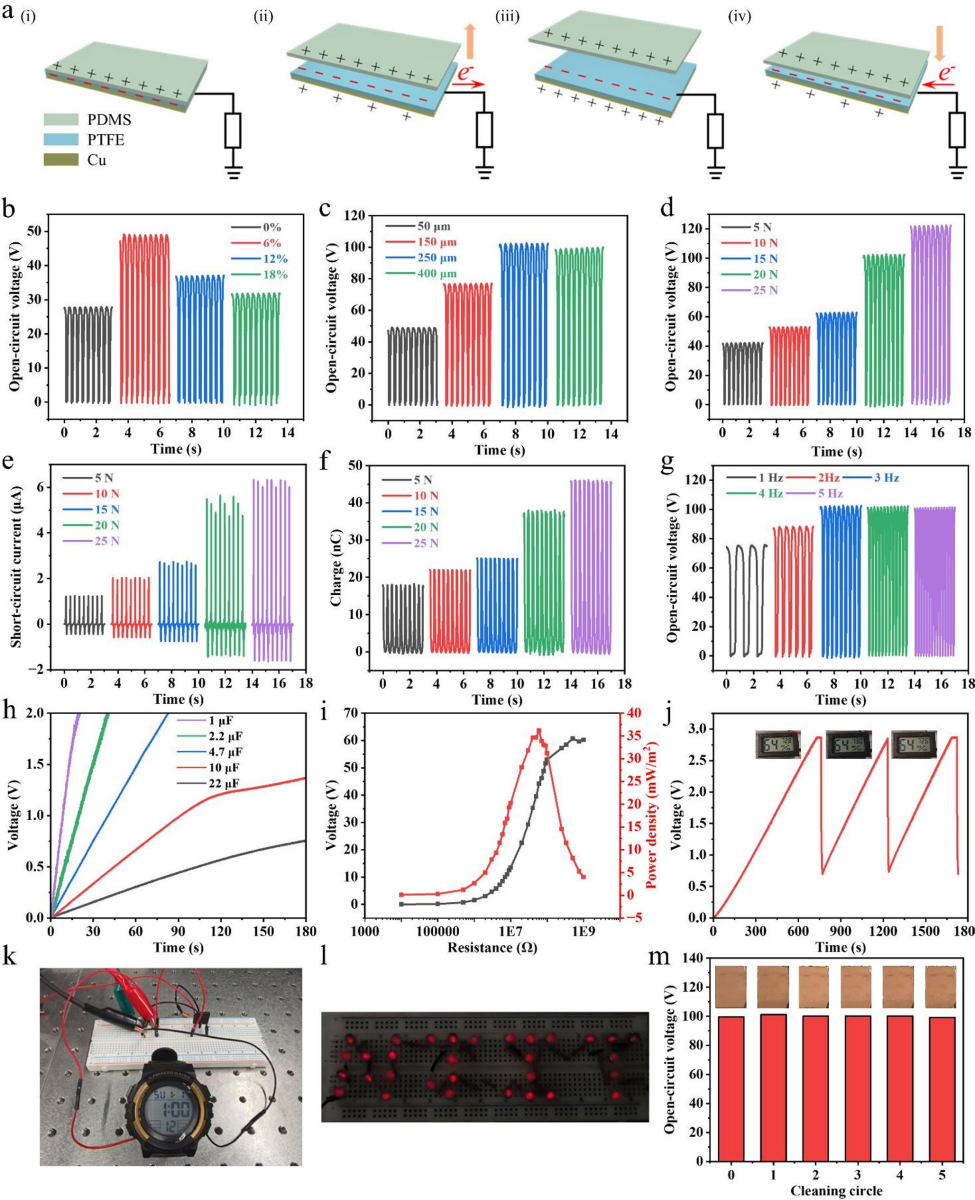
Output characteristics and energy harvesting performance of the PTFE-based TENG. (**a**) Schematic illustration of the working principle of the single-electrode PTFE-based TENG. (**b**) Open-circuit voltage of the PTFE-based TENG under different CNT loadings. (**c**) Effect of membrane thickness (with 6 wt% CNTs) on the output voltage of the PTFE-based TENG. (**d**) Open-circuit voltage, (**e**) short-circuit current, and (**f**) transferred charges of the PTFE-based TENG under varying applied forces. (**g**) Frequency-dependent open-circuit voltage characteristics of the PTFE-based TENG. (**h**) Charging curves of different capacitors powered by the PTFE-based TENG. (**i**) Output voltage and corresponding power density of the PTFE-based TENG under various external load resistances. (**j**) Charging and discharging behavior of a capacitor used to drive a digital thermo-hygrometer. (**k**) Continuous powering of a digital electronic watch enabled by a charged capacitor. (**l**) Instantaneous illumination of an LED array spelling “NJIT”. (**m**) Output stability of the PTFE-based TENG after repeated cleaning cycles under simulated contamination

Building on the above structure-property insights, we evaluated the device-level energy harvesting performance of a single-electrode PTFE-based TENG, systematically examining how CNT incorporation, membrane thickness, and key mechanical operating parameters translate into practical electrical outputs. The single-electrode PTFE-based TENG was constructed with a PTFE nanofiber membrane and a PDMS film with a rough surface as the triboelectric layers and copper as the electrode, and its operation principle is schematically illustrated in Fig. 3a. Periodic contact-separation between PDMS and PTFE generates triboelectric charges, driving the electron flow between the copper electrode and ground. As shown in Fig. 3b, incorporation of CNTs into the PTFE membrane significantly enhanced the PTFE-based output, with 6 wt% CNTs delivering the highest open-circuit voltage. To rationalize the CNT-loading-dependent output trend, we further analyzed the dielectric response and charge-dissipation tendency of the PTFE/CNT membranes (Figures S2-S4). Compared with pristine PTFE, introducing CNTs at 6 wt% increases the effective dielectric constant, indicating enhanced interfacial polarization and charge-storage capability, which favors the accumulation of triboelectric charges and thus boosts the output [36]. When the CNT content is further increased to 12 wt% and 18 wt%, the dielectric constant remains higher than that of the CNT-free PTFE membrane. Notably, the dielectric constant shows only a marginal increase from 12 wt% to 18 wt%, which is mainly attributed to CNT/PTFE agglomeration and entanglement that reduce the effective interfacial area and constrain further polarization enhancement. Nevertheless, the dielectric loss increases upon CNT incorporation and becomes more pronounced at higher CNT loadings (12 and 18 wt%), accompanied by a reduced impedance magnitude (Figures S3 and S4), indicating an elevated charge dissipation tendency. This enhanced dissipation can facilitate charge leakage via CNT-assisted charge transport, reducing the attainable surface charge density and lowering the open-circuit voltage. Therefore, 6 wt% CNTs provide an optimal balance between polarization-enhanced charge storage and suppressed leakage, whereas excessive CNT loading compromises the output due to increased charge dissipation.

To further optimize device performance, the effect of membrane thickness was further examined for PTFE membranes containing 6 wt% CNTs (Fig. 3c). Increasing the thickness from 50 to 250 μm progressively improved the output performance, as thicker membranes could accommodate more triboelectric charges with the assistance of CNTs. Nevertheless, further thickening slightly reduced the voltage, likely due to the diminished electrostatic induction between the electrode and the charges stored inside the membrane. Accordingly, a thickness of 250 μm was selected for subsequent studies. In addition, mechanical input conditions also played a critical role in the PTFE-based output, as shown in Fig. 3d. As the applied force increased, the output voltage rose from ~ 42 V to ~ 122 V, primarily due to the enlarged effective contact area between PTFE and PDMS, which led to increased triboelectric charge generation. The device exhibits an average sensitivity of 3.76 V kPa^− 1^, with notably enhanced responsiveness observed in the higher pressure region. Comparable force-dependent trends were identified in short-circuit current (Fig. 3e) and transferred charge (Fig. 3f). The influence of frequency was further investigated at a fixed force of 20 N. Increasing frequency initially led to slight increases in open-circuit voltage and transferred charge but eventually reached a plateau (Fig. 3g and Figure S5), reflecting the saturation of triboelectric charges at high frequencies. In contrast, the short-circuit current increased markedly with frequency (Figure S6), which is ascribed to the accelerated transfer of charges between the copper electrode and ground. Long-term operational stability was confirmed through durability testing under fixed contact-separation conditions of 20 N and 3 Hz. After more than 16,000 contact-separation cycles, the output remained stable (Figure S7), attesting to the robust performance of the device.

To further demonstrate the energy harvesting capability of the PTFE-based TENG device, charging tests were carried out using capacitors with different capacitances under the same actuation settings (20 N, 3 Hz). As shown in Fig. 3h, longer charging times were required for larger capacitances: a 4.7 µF capacitor was charged to 2 V within 90 s, while a 10 µF capacitor reached above 1.3 V in 180 s. The output characteristics under different external load resistances were also examined. In Fig. 3i, the voltage increased with load resistance and approached ~ 60 V, while the maximum power density of 36.18 mW m⁻² was achieved at 60 MΩ. In addition, the device was able to repeatedly power a digital thermo-hygrometer through a capacitor. Specifically, a 47 µF capacitor was rapidly charged to ~ 2.9 V under a mechanical input of 20 N at 3 Hz, sufficient to operate the thermo-hygrometer over successive charge-discharge cycles (Fig. 3j). Continuous powering of practical electronics was further validated, and the corresponding equivalent circuit is shown in Figure S8. As illustrated in Fig. 3k and Video S1, the PTFE-based device sustained a digital electronic watch for about 1 min after charging a 47 µF capacitor. Moreover, when connected to an array of 31 LEDs arranged to display the letters “NJIT”, the device instantly lit the entire array (Fig. 3l, Video S2), highlighting its potential for powering small-scale electronic systems.

Furthermore, the self-cleaning ability of the PTFE-based TENG was evaluated under a simulated heavy contamination condition, in which a thick layer of starch powder with an areal density of ~ 120 mg cm^− 2^ was spread on the membrane surface and subsequently rinsed with water. (Figure S9, Video S3). As a result, the starch contaminants were quickly removed, and the membrane surface remained dry. This superior self-cleaning action is attributed to the super-hydrophobic nature of the PTFE membrane surface, characterized by a high water contact angle (Fig. 2i). When water is applied, the contaminated particles are easily picked up and carried away by the rolling droplets without wetting or penetrating the membrane surface. Even after five cleaning cycles, the output performance of the PTFE-based TENG device was essentially maintained (Fig. 3m). Moreover, after self-cleaning under different starch areal densities, the output voltage showed no obvious variation (Figure S10), further confirming the reproducibility and robustness of the self-cleaning performance. This excellent self-cleaning property renders the PTFE nanofiber membranes highly fouling-tolerant, which is expected to significantly extend their service life, ensuring reliable operation of the PTFE-based TENG even under prolonged exposure to dust or environmental contamination. Overall, these results highlight the strong potential of the electrospun PTFE-based TENG for sustainable energy harvesting and its feasibility in powering small portable electronic devices. Notably, unlike conventional film-based harvesters that are prone to irreversible fouling, the PTFE-based TENG can operate in a fouling-tolerant mode enabled by an intrinsically super-hydrophobic, readily self-cleaning PTFE nanofiber membrane. Surface particulates can be removed by a simple manual rinse or natural precipitation, restoring performance without chemical solvents or reprocessing and thereby enabling low-maintenance deployment with broad prospects for field use in dust-rich or splash-prone settings.

### Breathable TENGs for self-powered sensing with high-temperature and underwater capability

**Fig. 4:**
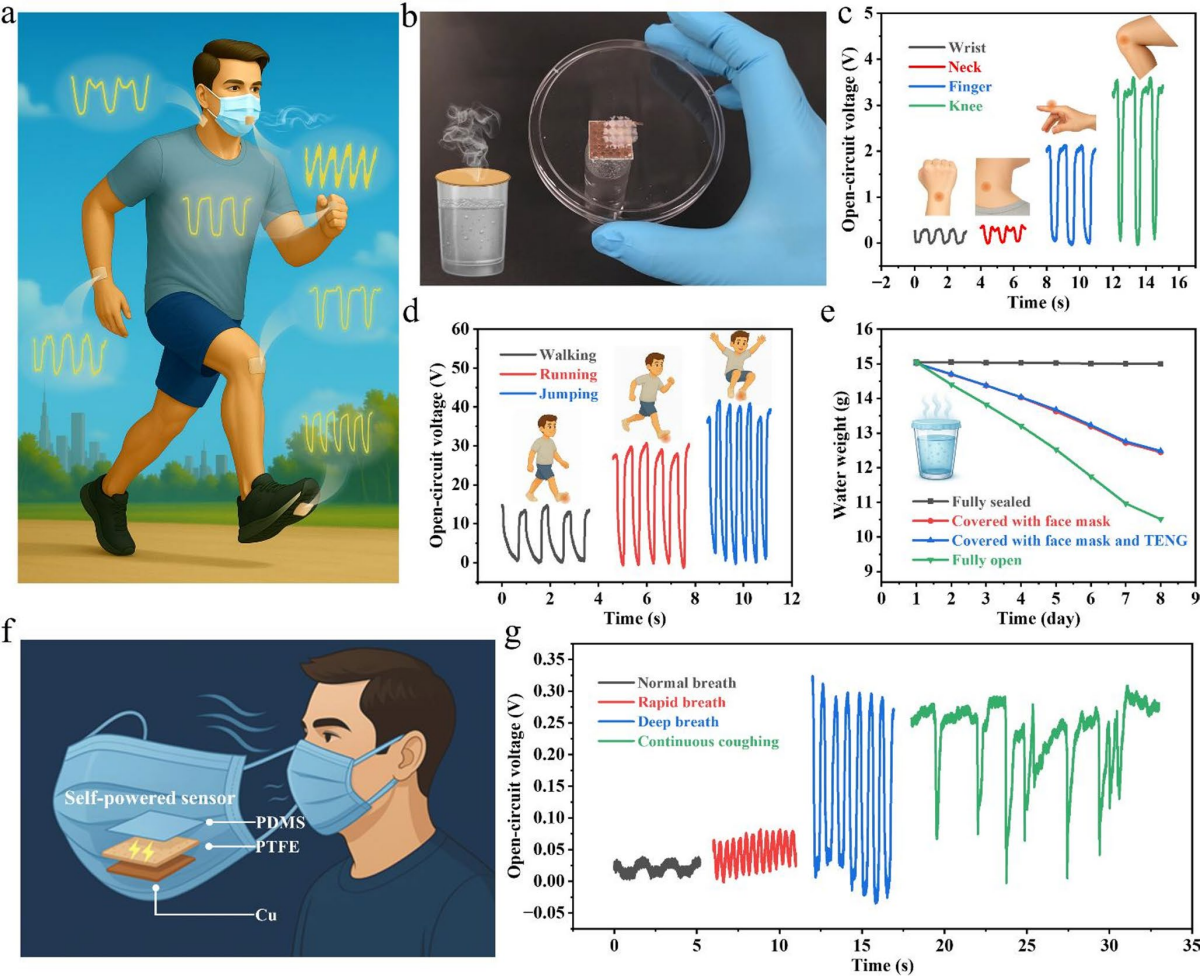
Motion monitoring and physiological sensing using the flexible and breathable PTFE-based TENG. (**a**) Schematic illustration of the motion monitoring and physiological sensing of PTFE-based TENG. (**b**) Breathability demonstration of the PTFE-based TENG. (**c**, **d**) Output voltage responses when the PTFE-based device is attached to different body parts (wrist, neck, finger, knee, foot). (**e**) Comparison of permeability among sealed, open, and face-mask covered conditions with and without the integrated TENG. (**f**) Schematic representation of the 2 × 2 cm² PTFE-based TENG integrated into a medical mask for respiratory monitoring. (**g**) Respiratory monitoring signals from the PTFE-based TENG, including normal, rapid, and deep breathing as well as continuous coughing

Motion detection and physiological monitoring play a vital role in health monitoring, where wearable sensors are indispensable for capturing real-time information and providing timely feedback. For such applications, comfort and breathability are particularly important, as the devices need to be seamlessly integrated with the human body. The soft and highly breathable electrospun PTFE nanofiber membrane developed in this study provides an ideal basis for constructing self-powered sensors. To further ensure permeability, a porous PDMS film was fabricated using a sacrificial template, and patterned porous copper electrodes were prepared by acutting process. The final device, a 2 × 2 cm² self-powered sensor comprising a PTFE nanofiber membrane, porous PDMS film, and a copper electrode, was designed for motion and respiration monitoring (Fig. 4a and Figures S11-S12). Given the importance of comfort and breathability for on-body applications, the device’s air permeability was first assessed before motion monitoring studies. As shown in Fig. 4b and Video S4, water vapor generated from hot water at 90 °C readily passed through the PTFE-based TENG, evidencing its outstanding breathability. The flexible sensor was subsequently attached to different body parts, including the wrist, neck, finger, and knee, where it exhibited stable conformability and reliably captured motion-induced signals (Fig. 4c). The distinct output patterns at each location highlight the versatility of the device in detecting joint movements of varying amplitudes and mechanical dynamics. Furthermore, the sensor effectively differentiated physical activities of different intensities. As illustrated in Fig. 4d, the output voltage increased from ~ 15 V during walking to ~ 30 V during running, and further to ~ 40 V during jumping, clearly reflecting both motion intensity and state transitions. This progressive signal amplification demonstrates the strong correlation between mechanical input and electrical response, thereby validating the device as an effective platform for self-powered multi-state human motion monitoring.

In addition to motion detection, the monitoring of physiological signals, particularly respiration with its rate and depth serving as vital health indicators, is also a critical aspect of health assessment. The integration of flexible sensors into masks provides a practical route toward real-time and non-invasive monitoring of breathing patterns, while also aligning with modern lifestyle habits, as mask wearing has become increasingly common in the contexts of public health and environmental pollution. Such integration holds substantial clinical value for patients requiring long-term respiratory observation, including those with asthma, chronic obstructive pulmonary disease (COPD), or sleep apnea. Motivated by these considerations, the flexible and breathable PTFE-based TENG developed in this work was incorporated into a medical mask to experimentally validate its feasibility for respiratory monitoring. To ensure user comfort, the effect of integration on mask permeability was first examined. As shown in Fig. 4e, a permeability comparison between medical masks with and without the PTFE-based TENG device revealed a negligible difference, confirming that the addition of the PTFE-based TENG sensor does not compromise breathability. As a result, the superior air permeability enables the TENG-integrated face mask to achieve real-time respiratory monitoring, where the self-powered sensor could detect airflow variations associated with different breathing behaviors (Fig. 4f). The device clearly captured respiratory behaviors of different rates and depths (Fig. 4g), offering an effective approach for routine breathing monitoring. In addition, distinct voltage peaks were also recorded during coughing, where the magnitude and frequency of the signals reflected the strength and rate of exhaled airflow. Such a capability could provide valuable information for diagnosing and tracking respiratory conditions of patients with respiratory diseases, thereby assisting physicians in making informed treatment decisions. Overall, these demonstrations highlight the potential of the PTFE-based TENG as a breathable, self-powered system for continuous monitoring of human motion and respiratory signals, opening promising opportunities for wearable health monitoring.

**Fig. 5:**
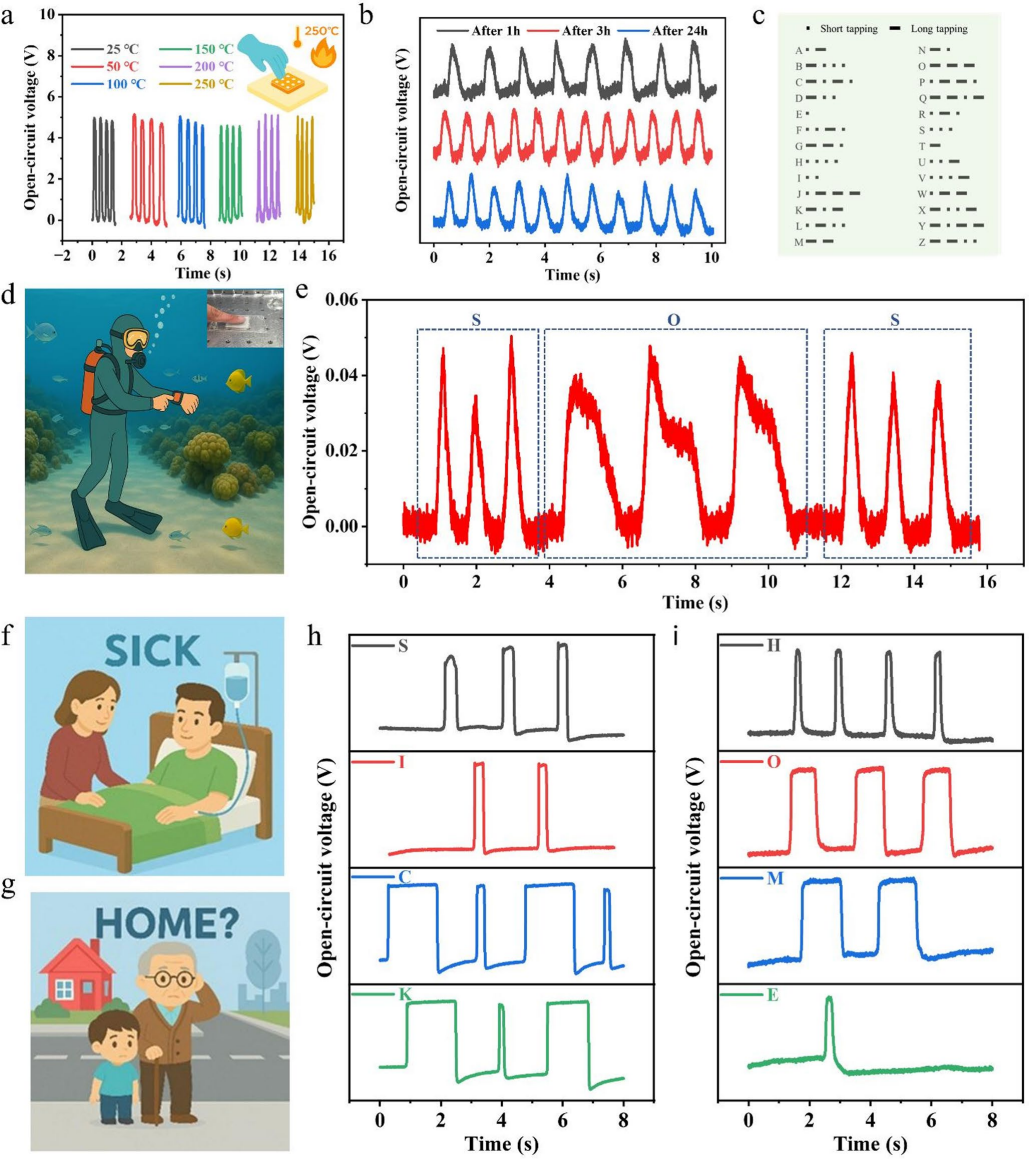
Environmental adaptability and multifunctional applications of the PTFE-based TENG. (**a**) Output stability of the TENG under different environmental temperatures (25–250 °C). (**b**) Open-circuit voltage of the TENG after soaking in water for 1 h, 3 h, and 24 h. (**c**) Encoding of Morse code (A-Z) through underwater finger tapping, where short and long voltage pulses represent “dots” and “dashes,” respectively. (**d**) Schematic illustration of potential application in diving suits for underwater emergency signaling. The inset depicts the underwater operation of the PTFE-based TENG. (**e**) Experimental demonstration of underwater “SOS” transmission after 24 h of immersion. (**f**, **h**) Practical application scenario for patients to transmit a “SICK” signal in medical settings. (**g**, **i**) Demonstration of “HOME” signaling for lost elderly individuals or children in outdoor environments

Beyond conventional conditions of motion and physiological signal monitoring, the environmental adaptability of the PTFE-based TENG was further investigated. Considering the excellent thermal stability of the PTFE nanofiber membrane, the output performance of the TENG was examined under elevated temperatures. As shown in Fig. 5a, stable open-circuit voltage signals were obtained from 25 °C to 250 °C under hand tapping with a gloved finger, demonstrating reliable high-temperature signal generation and remarkable resistance to thermal degradation. For a more controlled comparison, we further recorded the open-circuit voltage under the same mechanical actuation (5 N, 3 Hz) at different temperatures (Figure S13), where the waveform remained stable while the peak amplitude gradually decreased with increasing temperature. This attenuation is mainly attributed to accelerated charge relaxation at elevated temperatures, rather than structural failure of the PTFE membrane. In addition, stable short-circuit current signals were preserved during a durability test of over 6,000 cycles at 250 °C (Figure S14), further confirming robust high-temperature operation. Coupled with its intrinsic breathability, this high-temperature tolerance highlights the promising potential of the device for intelligent wearable applications in extreme thermal environments, such as integration into firefighter uniforms for real-time health monitoring and emergency signal transmission. In addition to high-temperature operation, the underwater capability of the PTFE-based TENG was also evaluated. To prevent electrode short-circuiting, the device edges were sealed with Tegaderm film before being subjected to underwater testing. The results in Fig. 5b reveal consistent and stable voltage outputs, evidencing reliable operation in aqueous conditions. This performance can be attributed to the intrinsic super-hydrophobicity of the PTFE nanofiber membrane, which prevents rapid water infiltration into the fibrous network and thereby preserves its triboelectric functionality. Notably, even after immersion for up to 24 h, the TENG continued to deliver consistent voltage signals, further confirming its robustness in underwater environments.

To further verify the underwater sensing signal generation capability of the PTFE-based TENG, finger tapping beneath the water surface was employed to simulate Morse code communication, where short-time finger contact produced transient voltage outputs corresponding to “dots,” and prolonged contact generated extended signals representing “dashes.” As a result, the complete set of alphabetic characters (A-Z) could be encoded through hand tapping, with information conveyed through specific sequences of short and long signals **(**Fig. 5c**)**. Considering the importance of underwater communication, such a capability is particularly valuable for divers, where the TENG could be integrated into diving suits to provide a simple means of transmitting emergency signals through finger tapping during hazardous operations (Fig. 5d). To experimentally validate this concept, the device was tested after immersion in water for 24 h and was still able to successfully transmit an “SOS” signal (Fig. 5e), confirming its robustness and applicability for underwater emergency communication. Beyond high-temperature and underwater applications, the PTFE-based TENG can also be employed in daily-life scenarios. For instance, in medical settings, patients may use the device to send a “SICK” signal to physicians (Fig. 5f, h). Similarly, in outdoor environments, elderly individuals or children who become lost could transmit a “HOME” signal to family members by rhythmically tapping the PTFE-based TENG (Fig. 5g, i), providing a simple yet effective means of communication in emergency situations. Overall, these demonstrations highlight the robustness of the PTFE-based TENG in extreme environments and adaptability to real-world applications, underscoring its strong potential for wearable electronics and emergency communication systems. To benchmark our device against recently reported breathable, hydrophobic, or high-temperature-resistant TENGs, a quantitative performance comparison is summarized in Table S1. Our CNT-reinforced PTFE-based TENG achieves a rare combination of high sensitivity, long-term durability, and stable operation up to 250 °C while maintaining super-hydrophobicity and breathability, highlighting its well-balanced performance relative to previously reported devices.

## Conclusion

In summary, we have developed a robust strategy to fabricate CNT-reinforced PTFE nanofiber membranes via electrospinning and sintering, enabling the construction of a fouling-resilient and multifunctional TENG capable of stable operation under diverse and harsh environmental conditions. The rational incorporation of CNTs enhanced the mechanical integrity and optimized charge storage and retention within the PTFE network, yielding significantly improved electrical outputs. The resulting membranes combined thermal stability, high permeability, and intrinsic super-hydrophobicity, thereby ensuring reliable operation under harsh environments. The PTFE-based TENG exhibited outstanding energy harvesting capability, effectively powering portable electronics such as a thermo-hygrometer, electronic watch, and LED arrays, while maintaining long-term durability and reliable recovery from contamination. Moreover, integration into wearable systems enabled effective monitoring of human motion and respiration without compromising breathability. The demonstrated adaptability under high-temperature and underwater conditions further underscores its potential for intelligent sensing and emergency communication. This work establishes a generalizable platform for constructing high-performance and environmentally resilient nanofibrous TENGs, offering a promising route toward next-generation self-powered systems for sustainable energy harvesting, wearable health monitoring, and adaptive electronics in extreme or contamination-prone environments.

## Experimental section

### Preparation of PTFE nanofiber membranes with different CNT loadings

PVA (Mw 89 000–98 000, 99+% hydrolyzed, Sigma-Aldrich) was dissolved in deionized water at 92 °C under magnetic stirring for 4 h. After cooling to room temperature, the PVA solution was combined with the aqueous PTFE dispersion (DISP 30, Fuel Cell Earth) and stirred for 1 h. Aqueous CNT (XFNANO Materials Tech) ink (0.033 wt%) was then introduced at mass fractions of 0, 6, 12, and 18 wt% relative to the aqueous PTFE dispersion, and the mixture was stirred at room temperature for at least 3 h until homogeneous. The detailed formulations of PTFE/PVA/CNT electrospinning solutions are summarized in Table S2. Electrospinning was carried out on a bench-top nanofiber electrospinning unit (MTI Corporation) equipped with a rotating mandrel collector, using an applied voltage of ~ 20 kV and a feed rate of 1 mL h^− 1^ under ambient conditions with a relative humidity of ~ 40%. The as-spun membranes were then detached and conditioned at ambient conditions for 12 h. Sintering was performed in a box furnace (Thermo Fisher Scientific) by ramping from room temperature to 300 °C at 3 °C min^− 1^ (dwell 10 min) and then to 375 °C at the same rate (dwell 20 min), followed by cooling to room temperature to obtain the PTFE nanofiber membranes with designated CNT loadings.

### Fabrication of PDMS films

Two types of PDMS films were prepared, including conventional micro-textured films and porous, breathable films. For the conventional film, PDMS base and curing agent (Dow Silicones Corporation) were mixed at 10:1 (w/w), hand-stirred for 5 min, and vacuum-degassed in a desiccator for ~ 30 min. A casting template was assembled by affixing a square 3D-printed PLA frame (QIDI Technology) onto a 1000-grit sandpaper sheet to impart surface texture. The degassed mixture was poured into the frame, leveled with a blade to ensure uniform thickness, cured at 80 °C for 30 min, and demolded to obtain the micro-textured PDMS film. For the porous film, the base and curing agent were first mixed and stirred at 10:1 (w/w) for 3 min. Sodium chloride (NaCl) granules were then incorporated at a PDMS: NaCl mass ratio of 1:2.2, followed by vigorous stirring for 10 min to achieve a homogeneous paste. After approximately 30 min of degassing, the mixture was cast in a PLA/sandpaper mold and leveled with a blade. Curing was performed at 80 °C for 1 h, after which the film was removed and leached in a 75 °C water bath for 8 h with at least three water exchanges to dissolve the salt. The film was finally dried at 80 °C for 4 h, yielding the porous, breathable PDMS films.

### Fabrication of PTFE-based single-electrode TENGs

Single-electrode TENGs operating in the contact-separation mode were assembled from PTFE nanofiber membranes, PDMS films, and copper foil tapes. For the energy harvesting configuration, a glass slide laminated with PI tape was used as the substrate. Square pieces (3 × 3 cm²) of copper foil tape and the PTFE nanofiber membrane were sequentially attached to the substrate. A conventional PDMS film of the same area served as the counter triboelectric layer for contact-separation. For the sensing configuration, a porous PDMS film, a PTFE nanofiber membrane, and a perforated copper foil tape measuring 2 × 2 cm² were stacked together to form the single-electrode TENG device. A 5 × 5 array of 2.8-mm circular holes was cut in the copper foil tape using a desktop cutting plotter (Silhouette America) to provide breathability.

### Material characterization

The microscopic morphology of PDMS films and PTFE nanofiber membranes with varying CNT loadings was observed through a field-emission scanning electron microscope (FE-SEM, JSM-7900 F, JEOL). The thermal behavior of the PTFE and PVA was characterized by a thermogravimetric analyzer (TGA 8000, PerkinElmer) and a differential scanning calorimeter (DSC 6000, PerkinElmer). Tensile experiments of the PTFE nanofiber membranes were performed using a digital force tester (Mark-10). To assess surface wettability, the static water contact angles of various PTFE nanofiber membranes were measured using a contact angle goniometer (Model 250, Ramé-hart Instrument). The dielectric constant, dielectric loss tangent, and impedance spectra of the PTFE nanofiber membranes were characterized by electrochemical impedance spectroscopy (EIS) using an electrochemical workstation (Reference 600+, Gamry Instruments). The electrical characterization of single-electrode TENG devices was carried out using a custom-built testing platform. Specifically, the mechanical excitation was supplied using an electrodynamic shaker (Model 2060E, The Modal Shop) driven by a waveform generator (33500B, Keysight Technologies) and a shaker amplifier (SmartAmpTM 2100E21, The Modal Shop). The electrical outputs were measured by an electrometer (Model 6514, Keithley Instruments).

### Self-cleaning test

To simulate dust contamination, a thick layer of starch powder was spread over the PTFE membrane surface. The sample was then mounted on a tilting stage at 35°. Deionized water was dispensed at the upper edge of the surface, and the removal of starch was recorded on video. The membrane was considered clean when no visible residue remained in the active area. The experiments involving human subjects have been performed with the full, informed consent of the volunteers, who are also the authors of the manuscript.

### High-Temperature electrical characterization

High-temperature electrical outputs of the TENG devices were evaluated on a temperature-controlled heating stage (Isotemp, Fisherbrand, Thermo Fisher Scientific). The device was fixed onto the heating stage using PI tape and positioned laterally, while magnetic blocks were used to secure the heating stage and prevent displacement during excitation. After the target temperature stabilized, as confirmed by the built-in controller and an infrared thermometer, the TENG was actuated either by cyclic contact-separation using an electrodynamic shaker or by manual pressing with a gloved finger at the target temperature. The electrical signals were recorded using an electrometer (Model 6514, Keithley Instruments).

### Underwater durability test

The TENG was placed on a glass slide and sealed along the device edges with Tegaderm films to isolate the electrode and lead connections from water and prevent short-circuiting during immersion. The glass slide was then fixed to the bottom of a transparent container, which was filled with deionized water. The device was manually pressed underwater, and the electrical outputs were continuously recorded using an electrometer (Model 6514, Keithley Instruments).

## Supplementary Information

Below is the link to the electronic supplementary material.

Supplementary Material 1

Supplementary Material 2

Supplementary Material 3

Supplementary Material 4

Supplementary Material 5

## Data Availability

The data that support the ﬁndings of this study are available from the corresponding author upon reasonable request.
