# Supplementary material for: Integrated carbon nanotube-reinforced PTFE nanofiber membranes for breathable, super-hydrophobic, and thermally resilient triboelectric nanogenerators
Source: Adv Compos Hybrid Mater. 2026 Feb 2;9(2):94. doi: 10.1007/s42114-026-01657-2 (PMC12909627; doi:10.1007/s42114-026-01657-2)
Supplement: Supplementary file 5 — Supplementary Material 5 [file 42114_2026_1657_MOESM5_ESM.docx]

**Supporting Information**

**Integrated Carbon Nanotube-Reinforced PTFE Nanofiber Membranes for Breathable, Super-hydrophobic, and Thermally Resilient Triboelectric Nanogenerators**

Yuxiao Wang, Lin Dong^*^

Department of Mechanical and Industrial Engineering, New Jersey Institute of Technology, Newark, NJ 07102, USA

***Corresponding author:**

Dr. Lin Dong: *lin.dong@njit.edu*


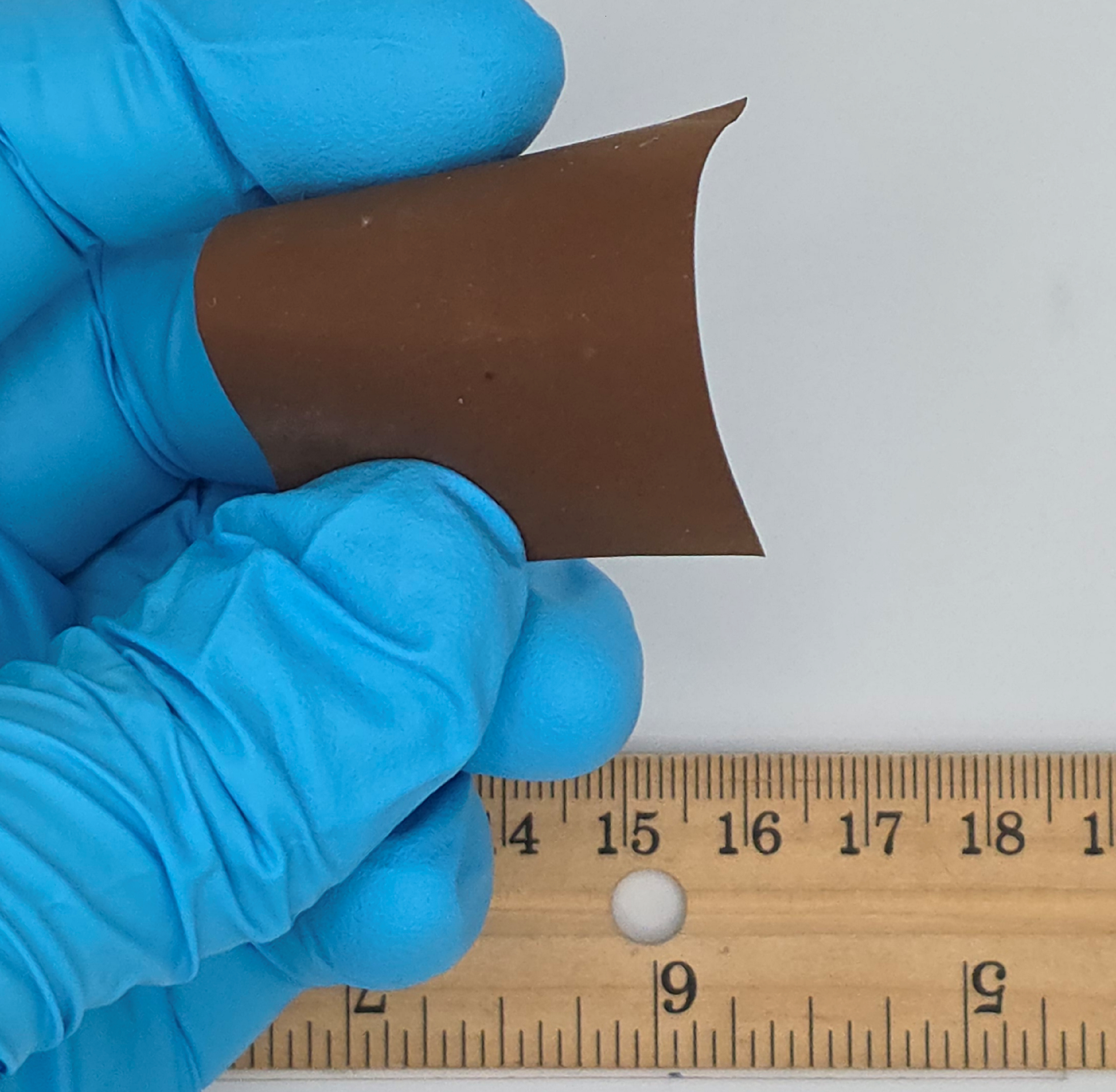


## Figure S1. Photograph of the as-prepared flexible CNT-reinforced PTFE nanofiber membrane.


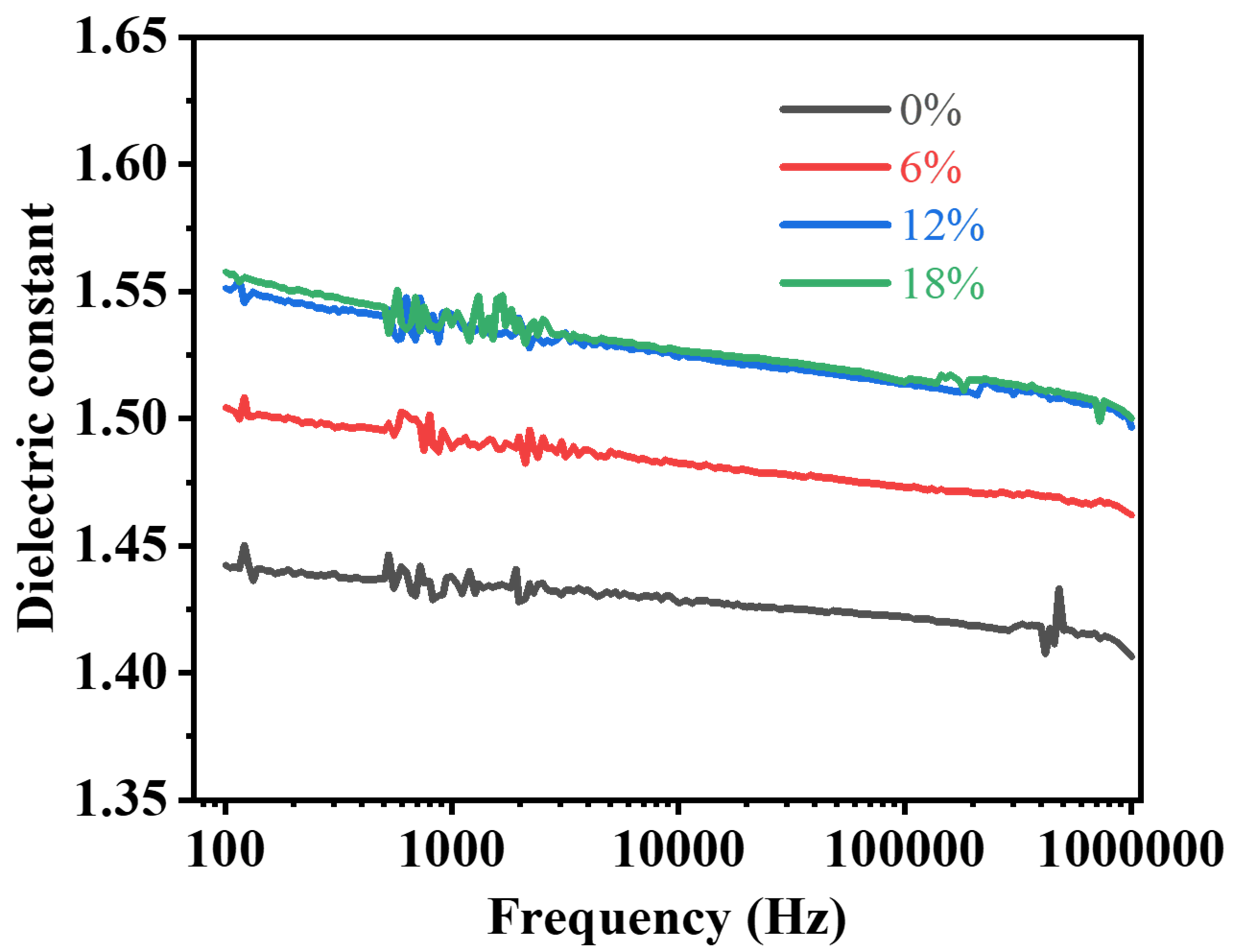


## Figure S2. Dielectric constant of PTFE nanofiber membranes with different CNT loadings.


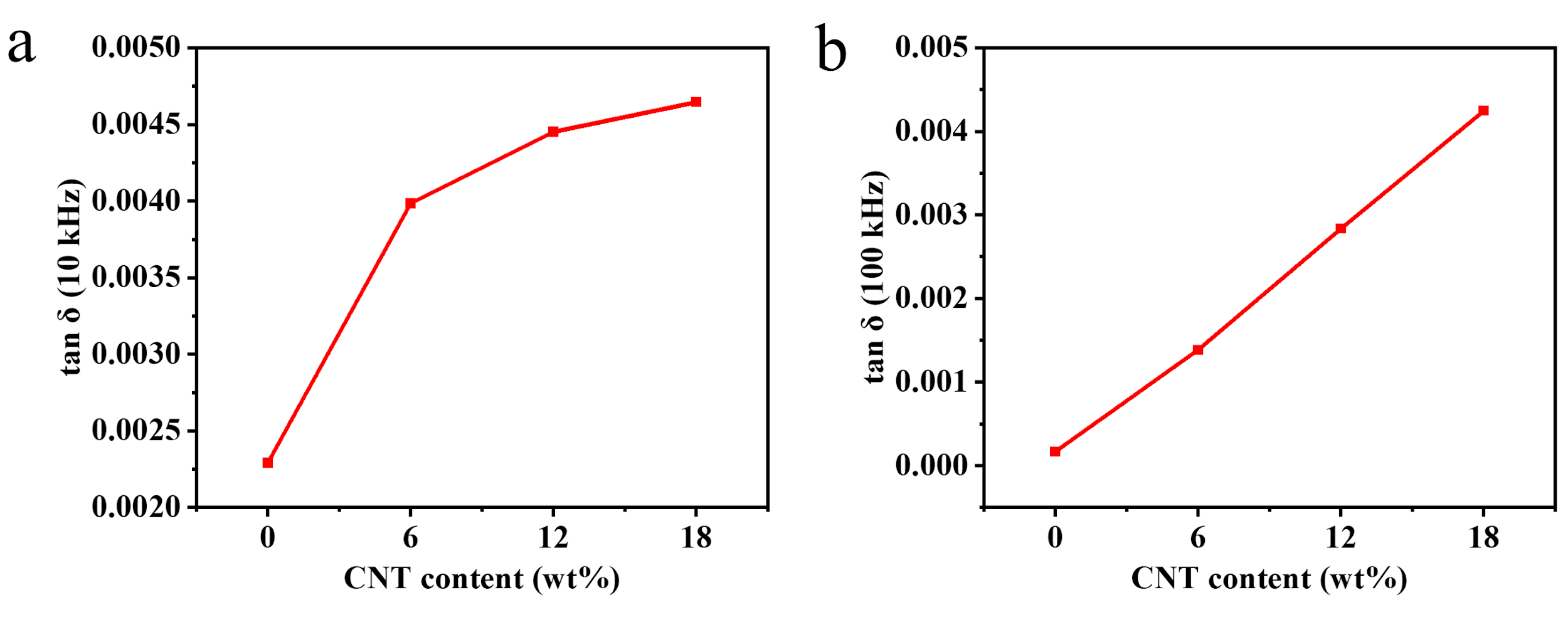


## Figure S3. Dielectric loss tangent (tan δ) of PTFE nanofiber membranes at 10 kHz (a) and 100 kHz (b) as a function of CNT content.


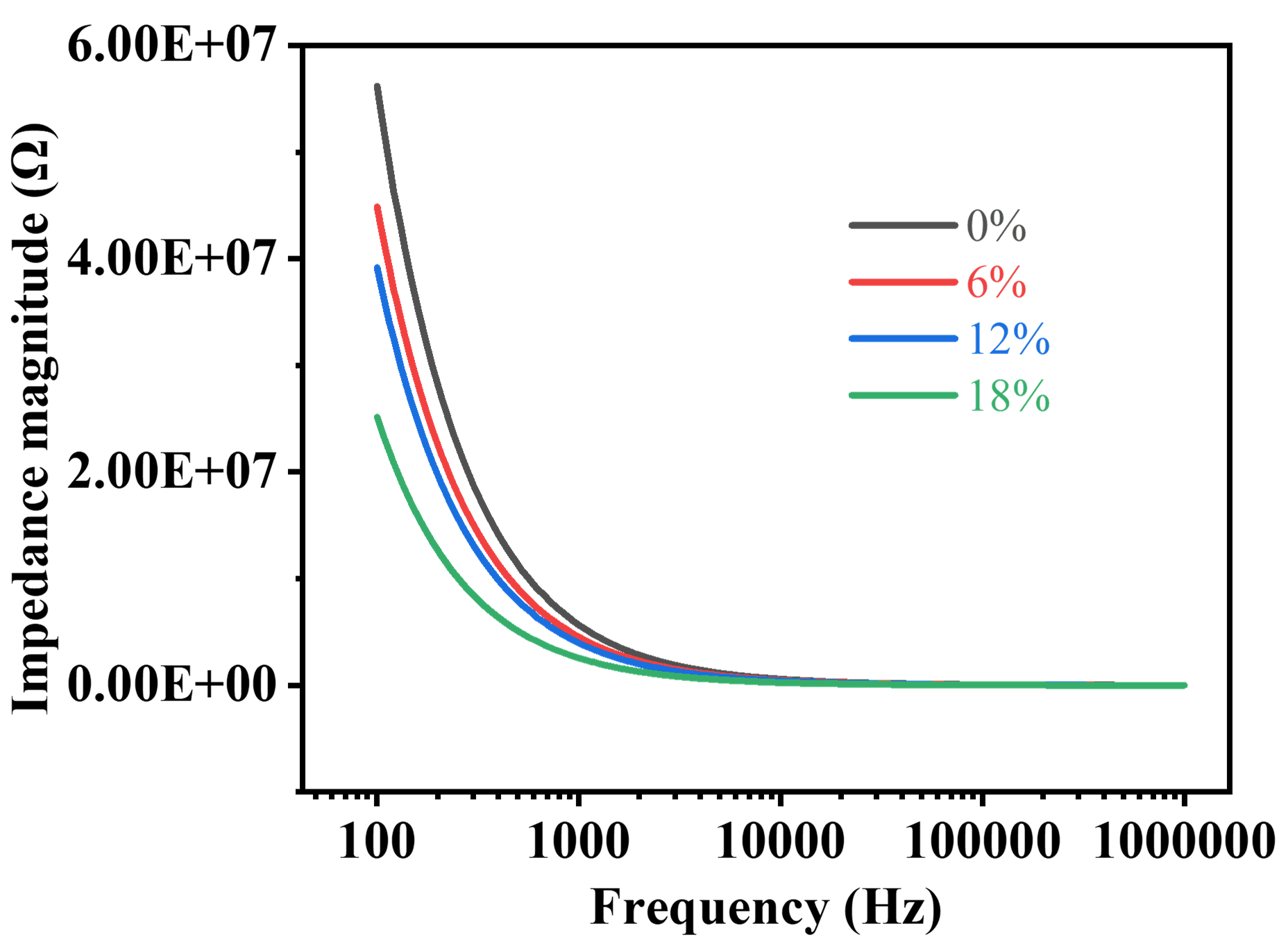


## Figure S4. Impedance magnitude of PTFE nanofiber membranes under different frequencies.


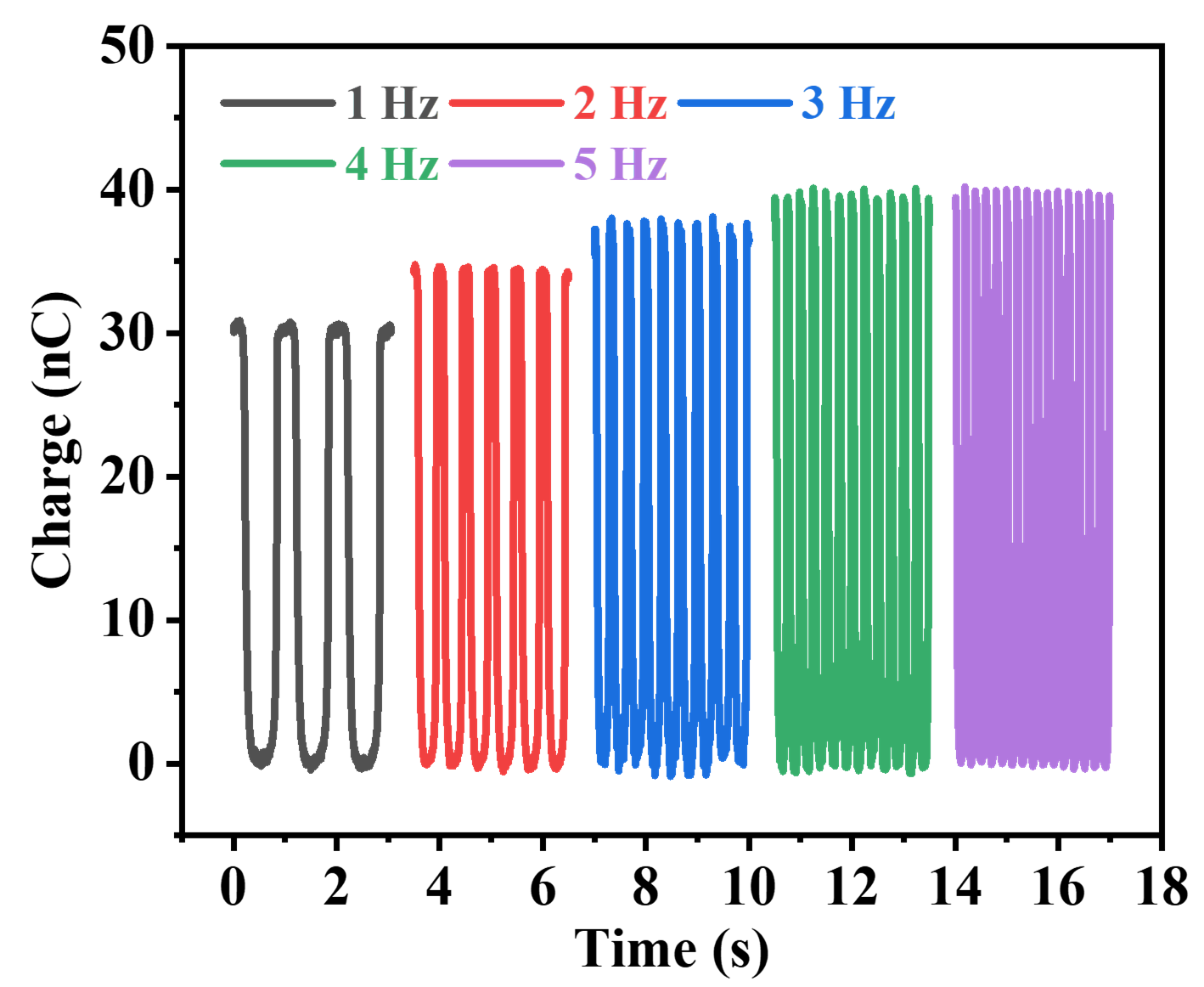


## Figure S5. Transferred charges of the PTFE-based TENG under different frequencies.


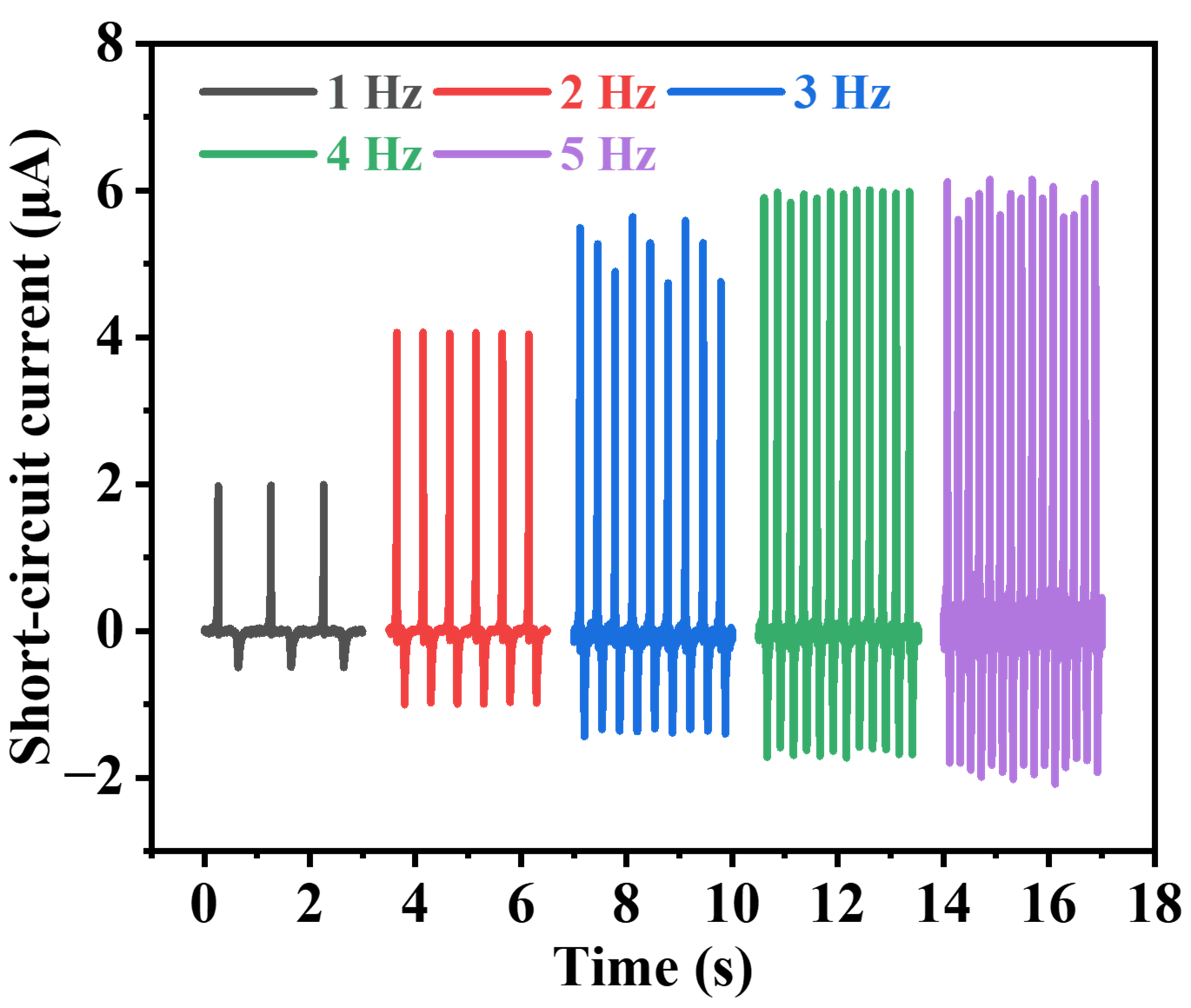


## Figure S6. Short-circuit current of the PTFE-based TENG under different frequencies.


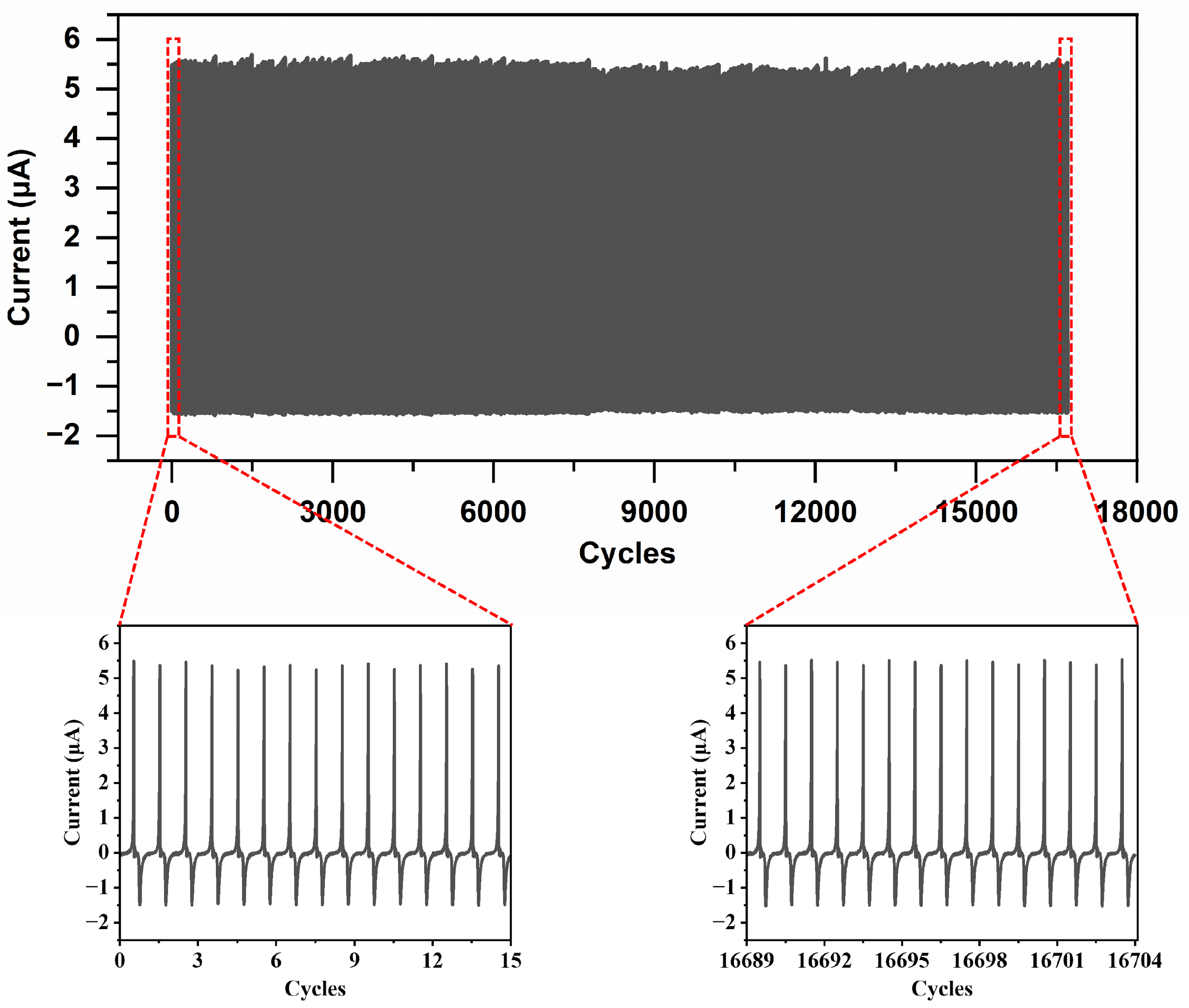


## Figure S7. The durability test of the PTFE-based TENG operating over 16000 cycles.


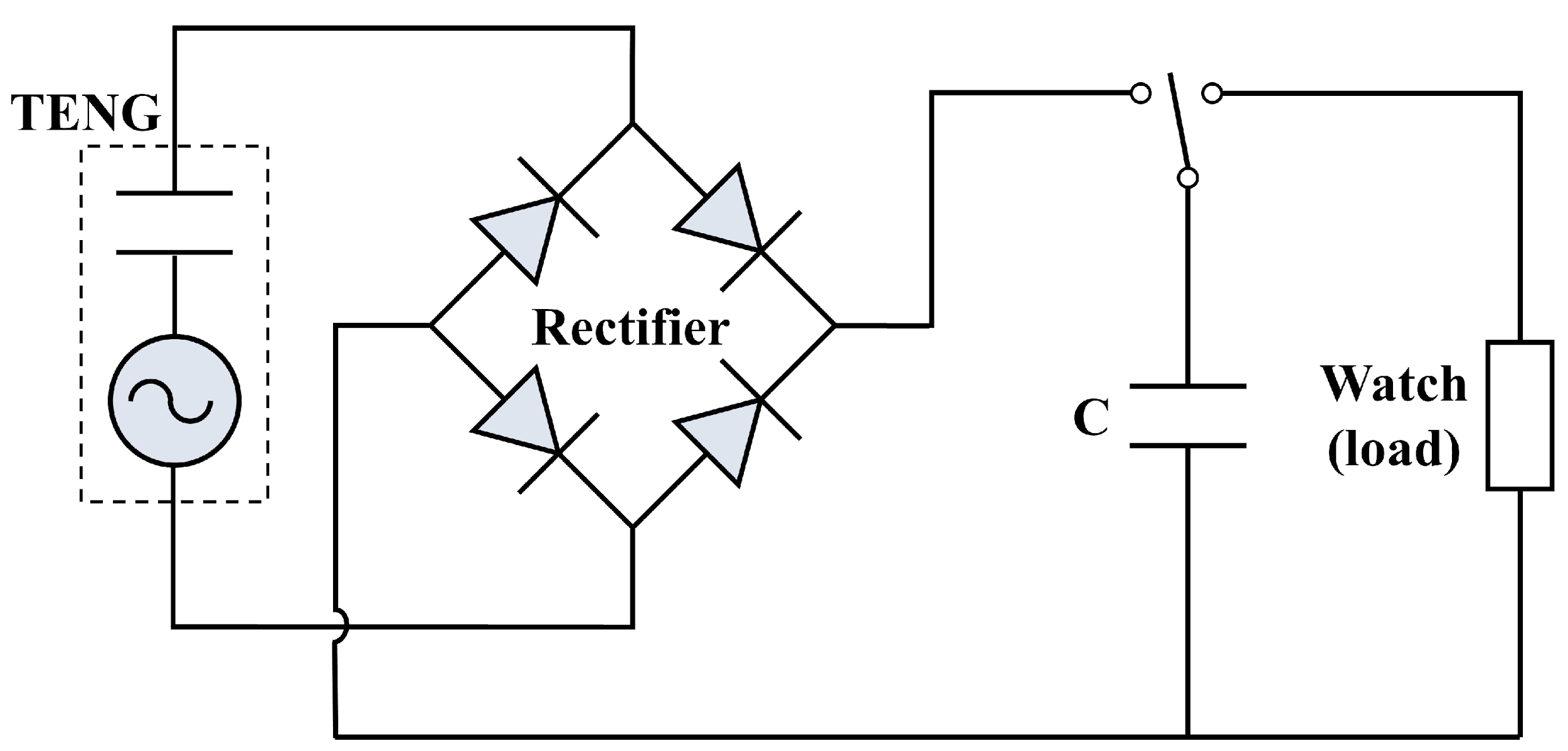


## Figure S8. An equivalent circuit illustrating capacitor charging and electronic watch powering by the PTFE-based TENG.

**
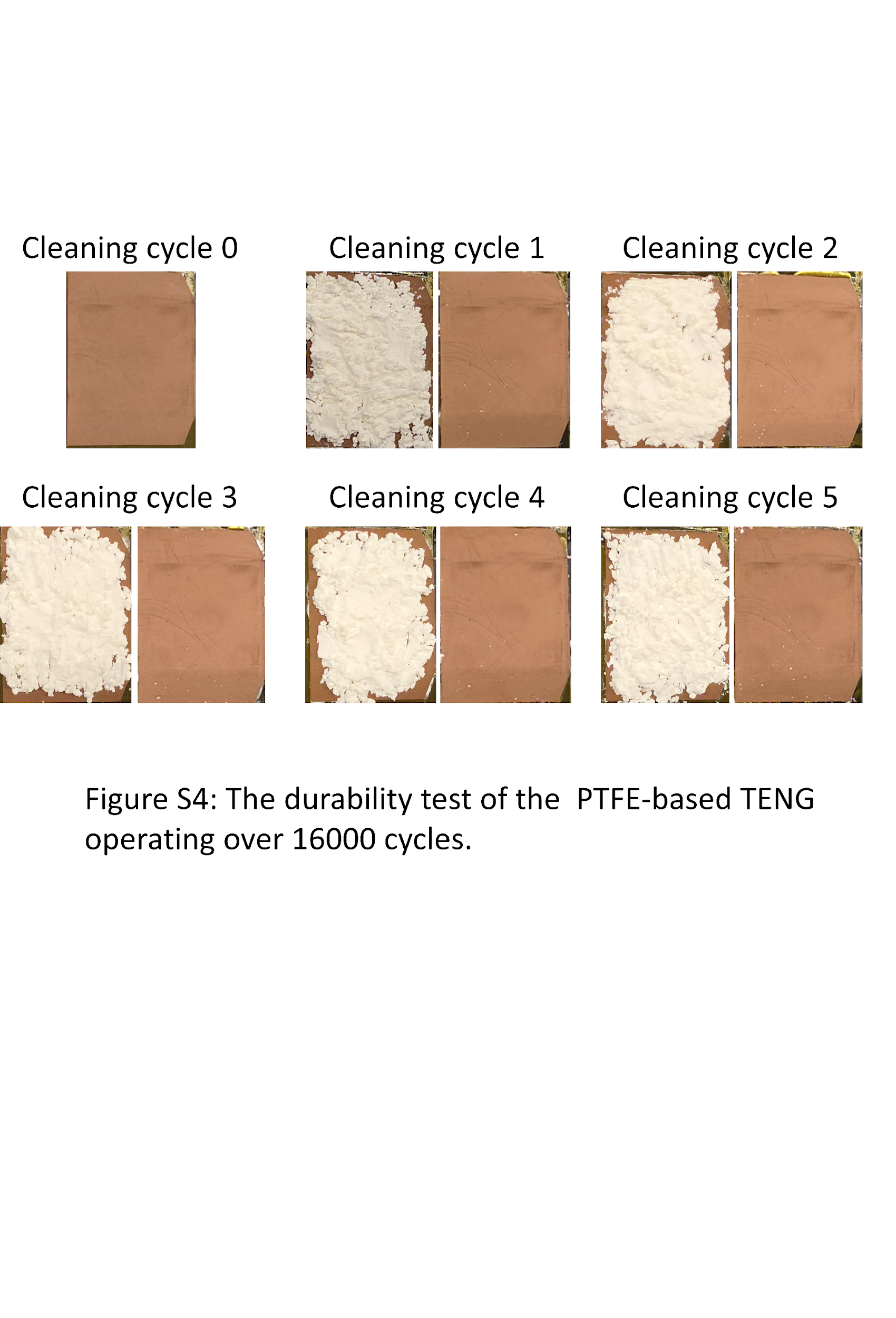
**

## Figure S9. Demonstration of the self-cleaning capability of the PTFE-based TENG under repeated contamination-cleaning cycles.


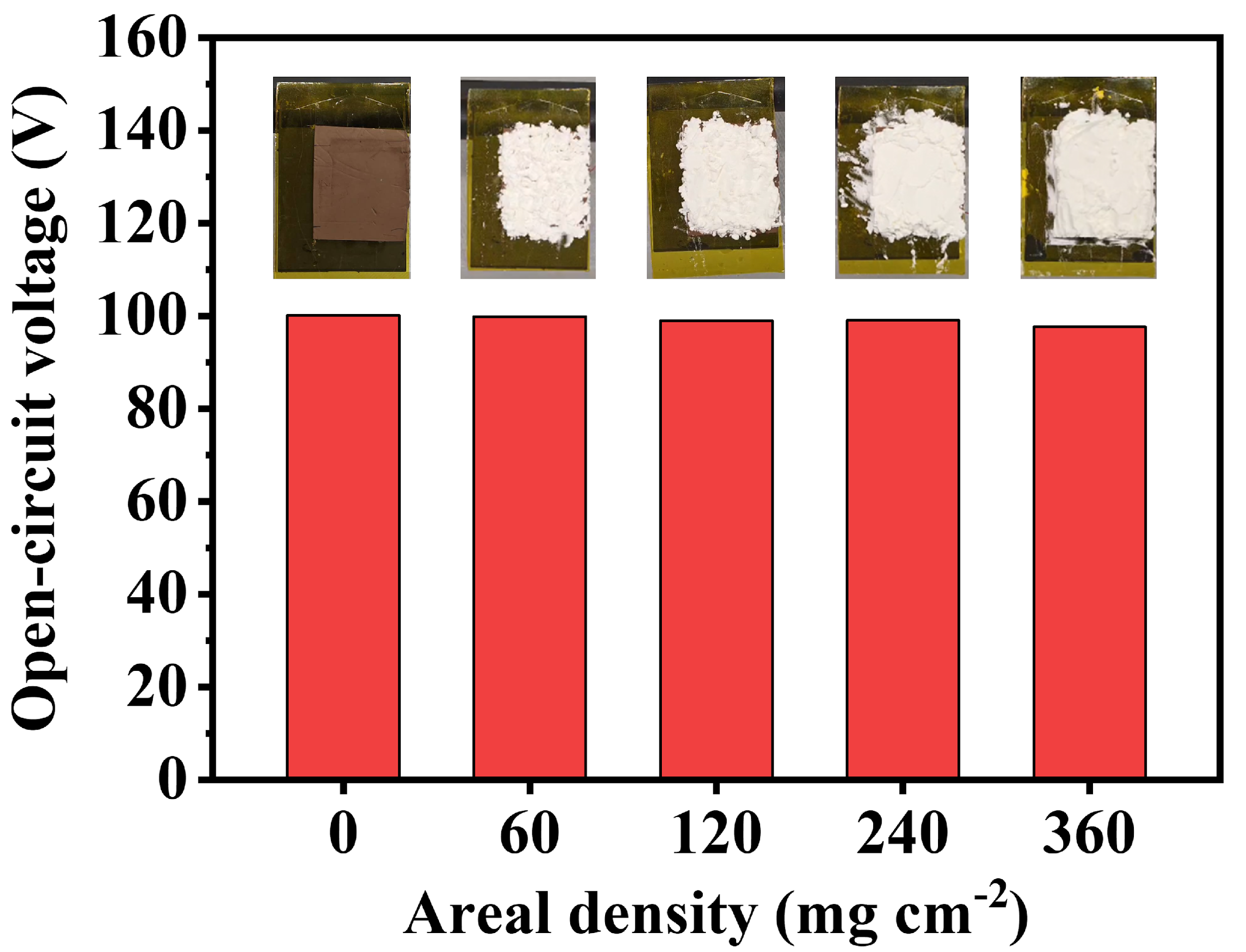


## Figure S10. Open-circuit voltage of the PTFE-based TENG after self-cleaning following starch contamination with different areal densities.


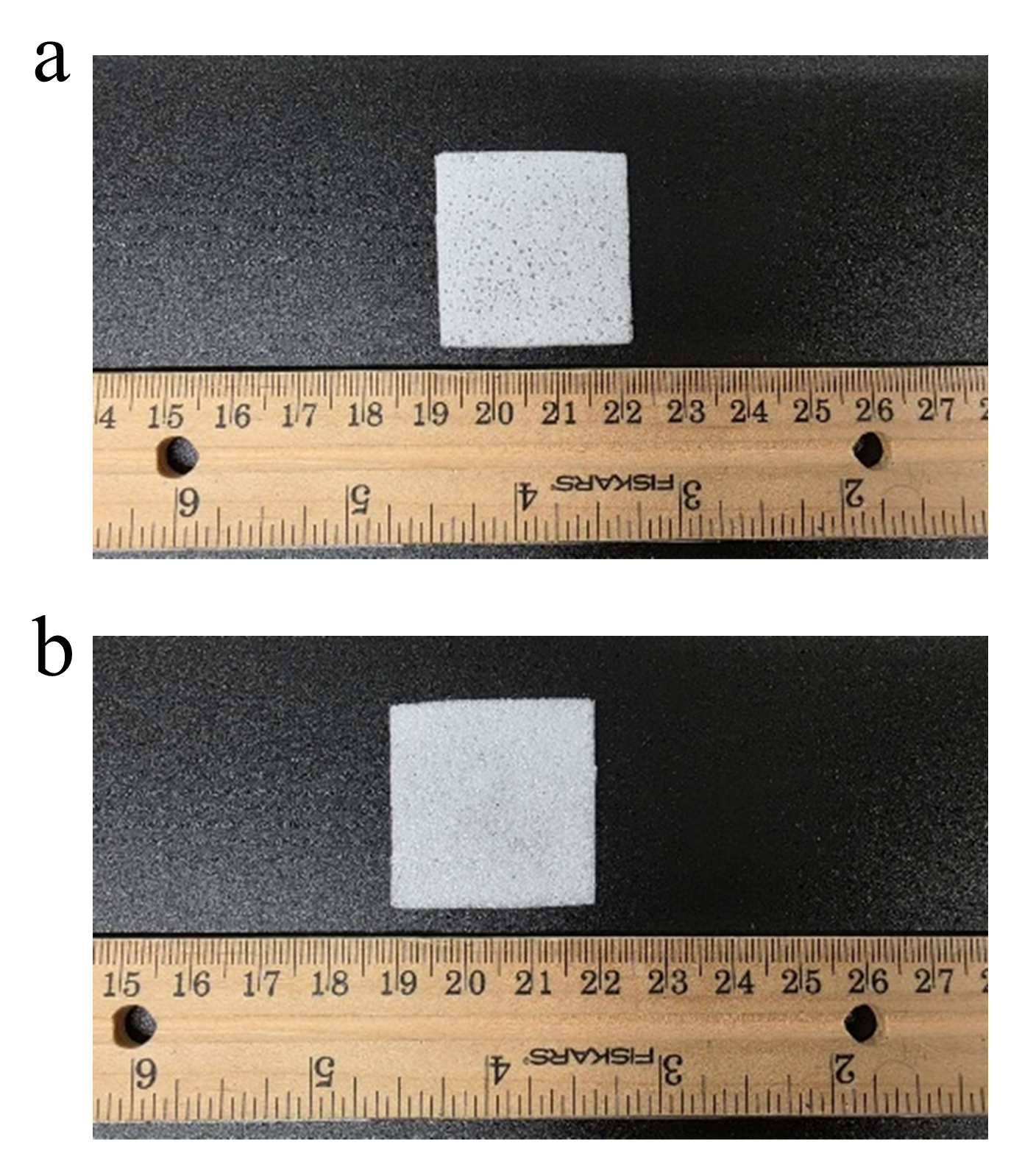


## Figure S11. Photographs of porous PDMS films. (a) The side in contact with the sandpaper substrate, serving as the friction interface with the PTFE nanofiber membrane in the TENG. (b) The side exposed to air.


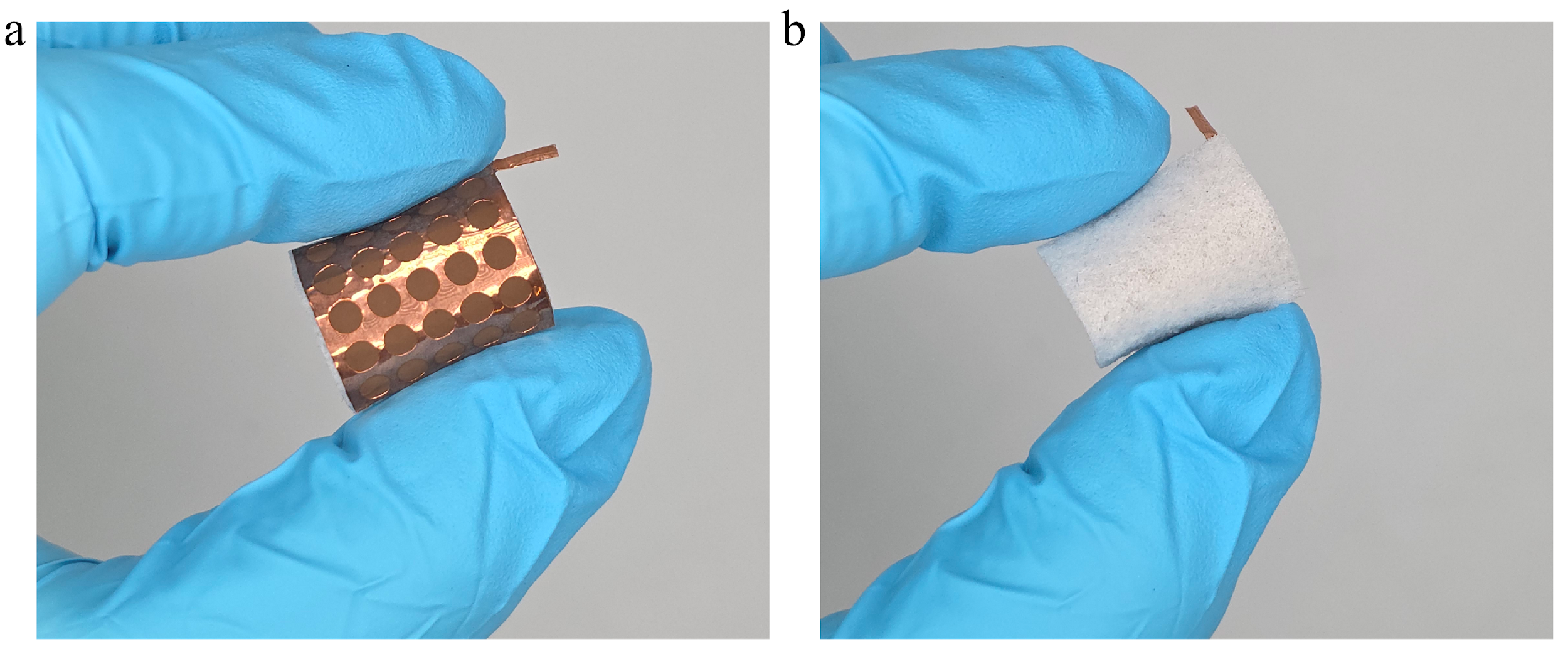


## Figure S12. Photographs of the two sides of the breathable TENG device. (a) Copper electrode/PTFE side. (b) Porous PDMS side.


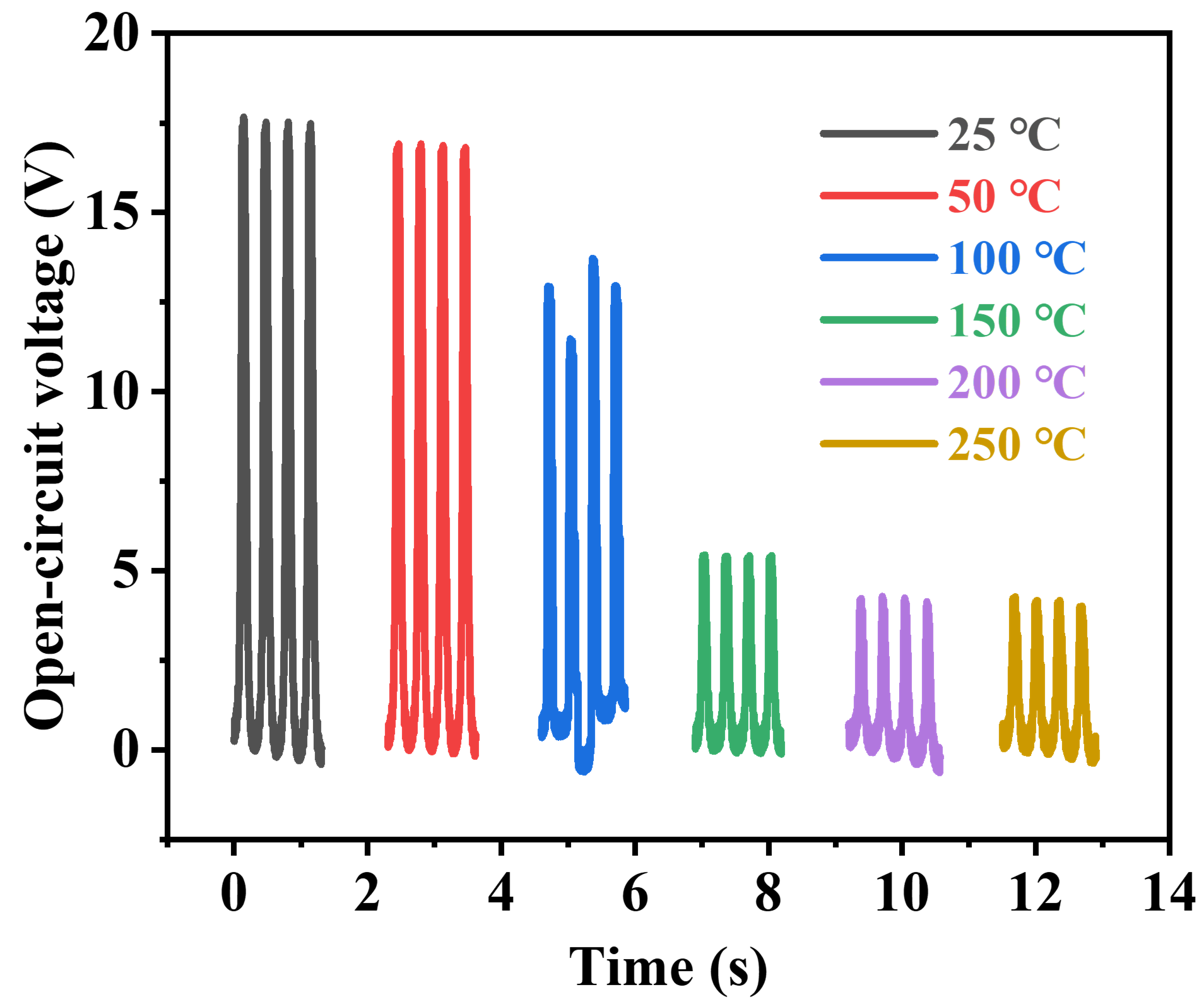


## Figure S13. Open-circuit voltage of the PTFE-based TENG measured at different temperatures (25-250 °C).


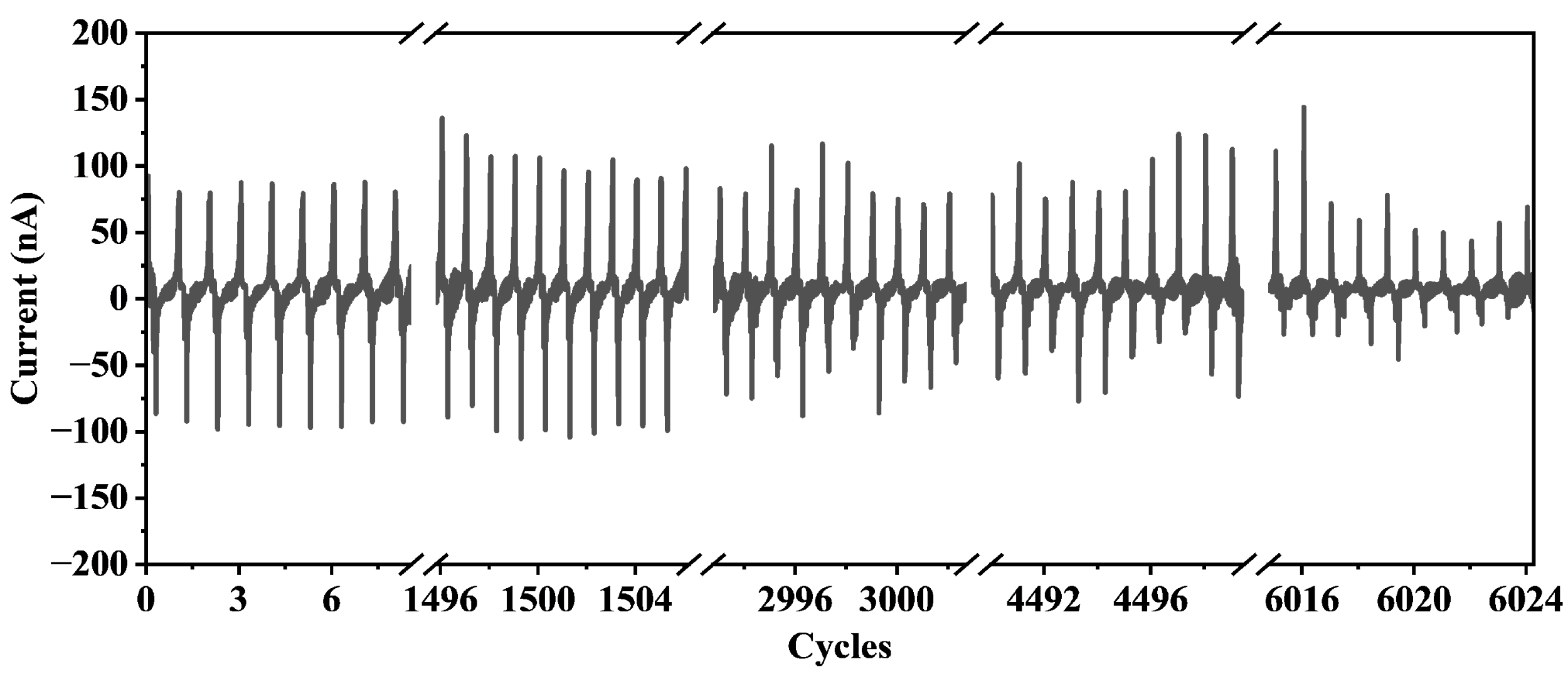


## Figure S14. Output stability of the PTFE-based TENG at 250 °C, showing short-circuit current signals recorded at selected cycle intervals during a durability test of over 6,000 cycles.

## Table S1. Performance comparison of this work with recently reported breathable, hydrophobic, or high-temperature-resistant TENGs.

| **Ref.** | **Max open-circuit voltage (V)** | **Sensitivity**  **(V kPa ^-1^)** | **Durability** | **Max working temperature (℃)** | **Water contact angle (°)** | **Breathability** |
| --- | --- | --- | --- | --- | --- | --- |
| [1] | 62.5 | 1.27 | / | 250 | 140.3 | Yes |
| [2] | 42 | / | 9000 | 200 | / | / |
| [3] | 124 | / | / | 100 | 88.2 | / |
| [4] | 110 | / | / | 120 |  | Yes |
| [5] | ~125 | / | / | 200 |  |  |
| [6] | 100 | / | / | / | 132 | / |
| [7] | 18.3 | / | / | / | 151 | / |
| [8] | ~7.5 | 8.36 | 10000 | / | 132.6 | Yes |
| [9] | 18 | / | / | / | 154 | / |
| [10] | ~37 | 0.16 | 12000 | / | / | Yes |
| [11] | 55 | / | 6000 | / | / | Yes |
| [12] | 20.3 | / | 3000 | / | / | Yes |
| [13] | 136 | / | 10000 | / | / | Yes |
| [14] | 20.1 | / | 10000 | / | / | Yes |
| **This work** | **122** | **3.76** | **16000** | **250** | **155.49** | **Yes** |

## Table S2. Formulations of PTFE/PVA/CNT electrospinning solutions.

| Sample | PVA (g) | Deionized water (g) | PTFE aqueous dispersion (g) | Aqueous CNT ink (g) |
| --- | --- | --- | --- | --- |
| PTFE/PVA | 0.54 | 3.46 | 3 | 0 |
| PTFE/PVA/  6 wt% CNT | 0.54 | 3.28 | 3 | 0.18 |
| PTFE/PVA/  12 wt% CNT | 0.54 | 3.1 | 3 | 0.36 |
| PTFE/PVA/  18 wt% CNT | 0.54 | 2.92 | 3 | 0.54 |

## References

[1] Hao M, Zhang X, Pan H, Hu X, Chen Z, Yang B, Liu Y, Gao X, Wang Q, Chen Z, Liu Y, Wang X, Liu Y. In-situ bonding enables robust fluorinated poly(imide-siloxane) based triboelectric nanogenerator for firefighter motion and location monitoring. *Nano Energy*. **2025**, *138*, 110856.

[2] Qiao X, Zhu L, Gao J, Yang L, Chen W, Chen C, Guan Q, You Z, Zhu M. Highly Robust and Intrinsic Flame-Retardant Polyphenylene Benzodiazole/Liquid Crystal Polyarylate Composites for Flexible Triboelectric Nanogenerators in Harsh Environment. *Adv Mater*. **2025**, e13780.

[3] Li J, Wu W, Li N, Wang N, Zhang X, Liu S, Wang M. Temperature-driven voltage enhancement in shape memory polymer triboelectric nanogenerators for high-temperature applications. *Chemical Engineering Journal*. **2025**, *523*, 168507.

[4] Chen Z, Zhou C, Xia W, Yin X, Wang Z, Fu X, Liu D, Lv J, Liu R, Peng Z, Song Y, Zheng L, Cai G. Strong, flexible and robust aramid nanofibers-based textile-triboelectric nanogenerators for high temperature escape monitoring and multifunctional applications. *Nano Energy*. **2024**, *123*, 109359.

[5] Song T, Jiang S, Cai N, Chen G. A strategy for human safety monitoring in high-temperature environments by 3D-printed heat-resistant TENG sensors. *Chemical Engineering Journal*. **2023**, *475*, 146292.

[6] Zhou L, Zhang D, Ji X, Zhang H, Wu Y, Yang C, Xu Z, Mao R. A superhydrophobic droplet triboelectric nanogenerator inspired by water strider for self-powered smart greenhouse. *Nano Energy*. **2024**, *129*, 109985.

[7] Zhang Q, Han Z, Yan J, Li S, Li C, Wu J, Li D, Zhang Y, Mai Z, Zhang Q, Zhang P. Enhanced induced charge in ramie-inspired triboelectric layer towards trace oil detection. *Nano Energy*. **2025**, *136*, 110711.

[8] Wang Y, Chu L, Meng S, Yang M, Yu Y, Deng X, Qi C, Kong T, Liu Z. Scalable and Ultra-Sensitive Nanofibers Coaxial Yarn-Woven Triboelectric Nanogenerator Textile Sensors for Real-Time Gait Analysis. *Advanced Science*. **2024**, 2401436.

[9] Lee G, Lee S, Kim D, Kim SH, Choi C, Lee SG, Cho K. Anisotropic Fluorinated‐Elastomer‐Blended Micro‐Dominoes for Wearable Triboelectric Nanogenerators. *Advanced Functional Materials*. **2024**, *34*, 2316288.

[10] Jiang Y, An J, Liang F, Zuo G, Yi J, Ning C, Zhang H, Dong K, Wang ZL. Knitted self-powered sensing textiles for machine learning-assisted sitting posture monitoring and correction. *Nano Research*. **2022**, *15*, 8389.

[11] Dong S, Xu F, Sheng Y, Guo Z, Pu X, Liu Y. Seamlessly knitted stretchable comfortable textile triboelectric nanogenerators for E-textile power sources. *Nano Energy*. **2020**, *78*, 105327.

[12] Peng Y, Huang H, Liu H, Dong J, Zhang Y, Long J, Huang Y. Robust Triboelectric E-Textile with Semi-bonded Bilayers for On-Skin Thermal Regulation and Self-Powered Motion Monitoring. *Advanced Fiber Materials*. **2025**, *7*, 1165.

[13] Liu R, Feng H, Sun Z, Cai Y, Qiu Y, Zhou R, Zhang Y, Xiao Y, Yang W, Weng M, Xu Y, Chen H. Enhancing output performance of triboelectric nanogenerator based on high-dielectric Ti3CNTx/PVA nanofiber membrane for human–computer interaction applications. *Chemical Engineering Journal*. **2025**, *519*, 165703.

[14] Hao Y, Zhang Y, Mensah A, Liao S, Lv P, Wei Q. Scalable, ultra-high stretchable and conductive fiber triboelectric nanogenerator for biomechanical sensing. *Nano Energy*. **2023**, *109*, 108291.
